# A multicenter noninferior randomized controlled study comparing the efficacy of laparoscopic versus abdominal radical hysterectomy for cervical cancer (stage IB3 and IIA2): study protocol of the LAUNCH 3 trial

**DOI:** 10.1186/s13063-023-07573-w

**Published:** 2023-08-18

**Authors:** Xin Wu, Hailin Yu, Yongrui Bai, Yanli Hou, Weihua Lou, Xipeng Wang, Tao Zhu, Yuyang Zhang, Weiguo Hu, Xiaohong Xue, Zhiling Zhu, Libing Xiang, Jiarui Li, Xuhong Fang, Shujun Gao, Hua Feng, Wenjing Diao, Hongwei Zhang, Ming Du, Weili Yan, Ling Qiu, Hao Feng, Shurong Zhu, Yan Du, Hua Jiang

**Affiliations:** 1 Obstetrics and Gynecology Hospital, Fudan University, 128 Shenyang Rd 200090, Shanghai, China; 2 Shanghai Jiao Tong University School of Medicine, Renji Hospital, Shanghai, China; 3 Xinhua Hospital Affiliated to Shanghai Jiao Tong University School of Medicine, Shanghai, China; 4 Cancer Hospital of the University of Chinese Academy of Sciences (Zhejiang Cancer Hospital), Hangzhou, China; 5 The First Affiliated Hospital of Wenzhou Medical University, Wenzhou, China; 6 Zhongshan Hospital Affiliated to Fudan University, Shanghai, China; 7 Children’s Hospital of Fudan University, Shanghai, China

**Keywords:** Cervical cancer, Stages IB3 and IIA2, Laparoscopic radical hysterectomy, Abdominal radical hysterectomy, Randomized controlled trials, Overall survival, Progression-free survival, Prognosis

## Abstract

**Background:**

Cervical cancer is and will remain to be an important health problem in China, especially with an increasing proportion of younger patients who has more specific needs. In China, surgery to remove tumor burden followed by postoperative treatment with radiotherapy and chemotherapy based on clinicopathologic factors may be the best choice for stages IB3 and IIA2 patients. Radical hysterectomy in cervical cancer has been a classic landmark surgery in gynecology. The current trial is designed to evaluate whether there is a difference between laparoscopic RH and abdominal RH in cervical cancer (stages IB3 and IIA2) patient survival under stringent operation standards and consistent surgical oncologic principles. This paper reports the rationale, design, and implementation of the trial.

**Methods/design:**

This is an investigator-initiated, prospective, randomized, open, blinded endpoint (PROBE) controlled trial. A total of 1104 patients with stage IB3 and IIA2 cervical cancer will be enrolled over a period of 3 years. Patients are randomized (1:1) to either the laparoscopic RH or the abdominal RH group. Patients will then be followed up for at least 5 years. The primary end point will be 5-year overall survival, and secondary endpoints include 5-year progression-free survival, recurrence, and quality of life measurements.

**Discussion:**

The study results will provide more convincing evidence-based information for stages IB3 and IIA2 cervical cancer patients and their gynecologic cancer surgeons in their choice of surgical method.

**Trial registration:**

ClinicalTrials.gov, NCT04939831, retrospectively registered on 25 June 2021.

**Supplementary Information:**

The online version contains supplementary material available at 10.1186/s13063-023-07573-w.

## Background

Cervical cancer is the most common malignant tumors of female reproductive system in the world [1]. For early-stage (including IA1 with lymphovascular space invasion (LVSI), IA2, IB1, IB2, IIA1; and IB3 and IIA2) cervical cancer patients, radical surgery is the main treatment procedure, which is more difficult to perform and may possibly lead to more complications due to larger surgical margin [2]. Compared with traditional abdominal radical hysterectomy (ARH), minimally invasive radical hysterectomy has unique advantages, such as less abdominal wall trauma, less pain, clearer vision and less bleeding, less interference with the intestine, and lower postoperative infection rate. Modern standard surgery includes laparoscopic surgery (divided into conventional laparoscopic surgery and robotic-assisted laparoscopic surgery, or multiport laparoscopic surgery and single-port laparoscopic surgery), open surgery, and transvaginal surgery. Laparoscopic surgery and open abdominal surgery can achieve the same range of resection, it is intuitively assumed that they achieve similar results, and therefore most previous studies have focused on comparing their operative time, intraoperative bleeding, length of hospital stay, and cost, rather than survival indicators such as progression free survival (PFS) and overall survival (OS). Only a few small studies have evaluated the survival benefit of LRH for cervical cancer: in terms of PFS [3–8], LRH was not inferior to ARH, while laparoscopy was superior to open surgery in terms of operative time, intraoperative bleeding, and length of hospital stay.

In 2018, the New England Journal of Medicine published two studies that compared survival results between abdominal and minimally invasive radical surgeries for early-stage cervical cancer patients [9, 10]. Both the retrospective cohort analysis [9] and the randomized clinical trial (RCT) of the Laparoscopic Approach to Cervical Cancer (LACC) study [10] have suggested that the minimally invasive radical cervical surgery group is associated with both lower disease-free survival (DFS) and OS rates compared with the open surgery group for early-stage cervical cancer patients. The above results have caused a huge impact on the gynecology community. As a result, the National Comprehensive Cancer Network (NCCN) guidelines (version 3.2019) have recommended open surgery as the standard procedure for cervical cancer. However, the cohort study included patients with stage IA2 or IB1 cervical cancer, and the LACC trial included stage IA1, IA2, and mostly IB1 (about 92%) cervical cancer patients.

According to the International Federation of Gynecology and Obstetrics (FIGO) and NCCN guidelines, concurrent chemoradiotherapy (CCRT) is the first treatment choice for stages IB3 and IIA2 cervical cancer patients. For these patients, postoperative radiotherapy and chemotherapy are usually required after radical hysterectomy [11]. However, this multi-modal treatment increases the occurrence of complications with limited survival benefits, which is the main reason of both FIGO and NCCN guidelines recommending CCRT as the preferred treatment for stage IB3 and IIA2 cervical cancer patients, while the FIGO guideline also emphasizes that the choice of treatment strategy should base on comprehensive evaluation of available resources, patient and tumor conditions. In China, high-quality radiotherapy resources are unevenly distributed and are not universally accessible. Under this circumstance, surgery to remove tumor burden (intraoperative optimization process to ensure tumor-free principles), followed by postoperative treatment with radiotherapy and chemotherapy based on clinicopathologic factors, is the best choice for those patients. In addition, some cervical cancer with large lesions, especially adenocarcinomas, are not sensitive to radiotherapy [11]. What is more, a certain proportion (8.5–12.6%) of cervical cancer patients is younger than 35 years [12]. For young patients with stages IB3 and IIA2 cervical cancer, if CCRT is used, vaginal function is lost and ovarian function also declines after concurrent radiotherapy and chemotherapy, thus seriously affecting subsequent life quality. However, if radical hysterectomy is chosen, ovarian suspension can be performed during the operation to reduce the side effect of postoperative radiotherapy.

To address these problems, we designed the current RCT study to compare outcomes of stages IB3 and IIA2 cervical cancer patients receiving laparoscopic RH vs. abdominal RH under consistent surgical oncologic operating regulations.

## Methods/design

### Trial design

**Fig. 1:**
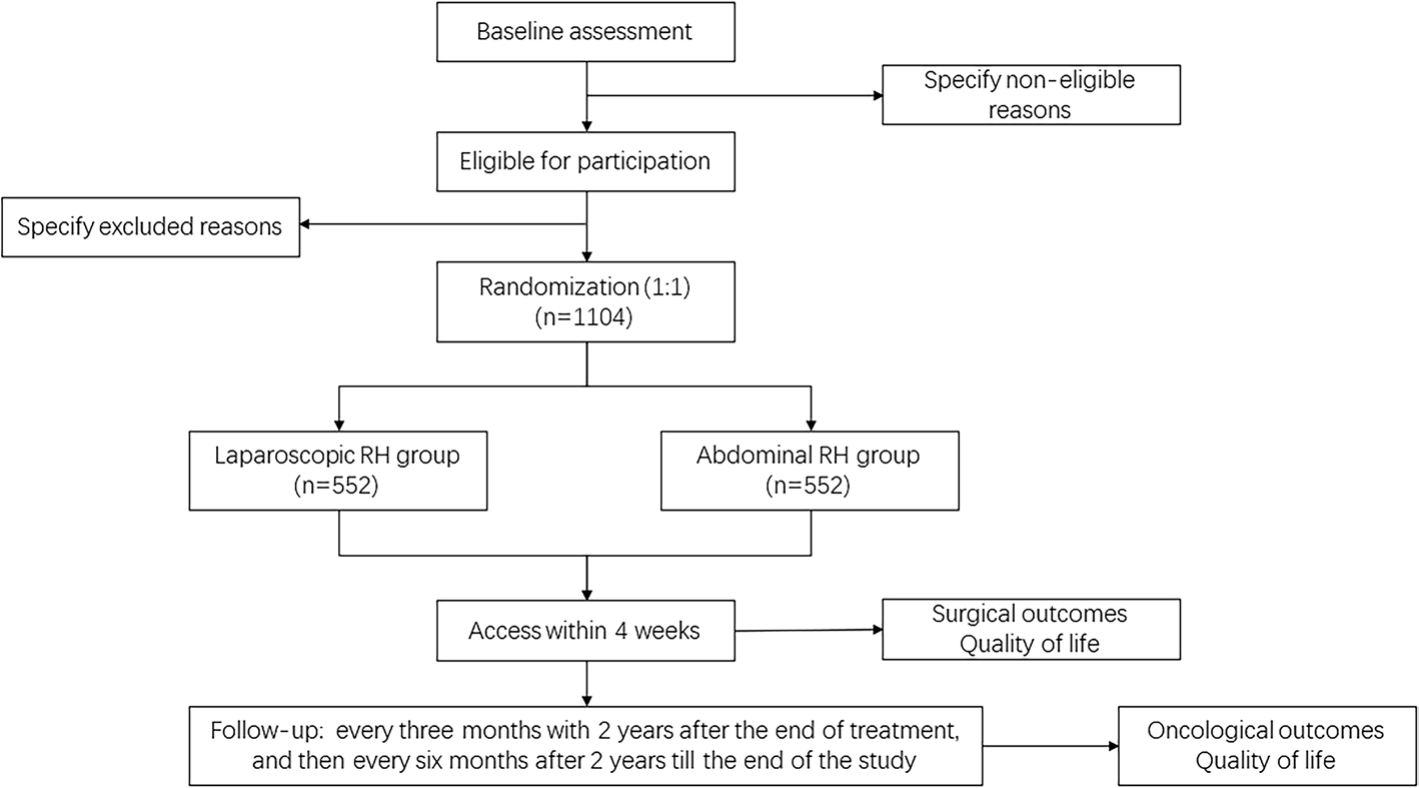
Study flow diagram

This trial is the third study of the LAUNCH trial series [13, 14]. It is an investigator-initiated, prospective, randomized, open, blinded endpoint (PROBE) controlled non-inferiority trial. Eligible patients are randomly assigned to receive either abdominal or laparoscopic RH (Fig. 1). The study protocol follows the SPIRIT statement for clinical trial protocols [15] and the SPIRIT-PRO Extension [16].

A trial steering committee (TSC) is established to provide oversight of the study conduct, patient safety, data collection, and statistical analysis. TSC members include principal investigators (PIs) at the main center, co-PIs at sub-centers, and the study statistician. The TSC will meet semi-annually to review study progress, inspect data quality, draft reports, and discuss any necessary modifications. Protocol modifications will be exchanged to relevant parties by the PIs after agreement by the steering committee. TSC will also provide related information for other committees. At the main center, a trial coordinating center which includes dedicated research staff is set up to provide daily support for running the trial. The main purpose of the coordinating center is to ensure consistent study quality across each center, and its functions include scheduling relevant meetings, collecting and releasing information, receiving feedback from each center, and handling emergency situations during the study process. The independent data monitoring committee (DMC) is consisted of a gynecologist, an epidemiologist, and a statistician, which is to oversee patients’ safety and trial quality. The DMC has met and established a charter and will have annual meetings. The attending gynecologist will record and report adverse events (AEs) during the study according to the charter. All AE data will be reviewed and assessed by the DMC, which will make recommendations to the TSC if the protocol needs revision or the trial should be discontinued (Additional file 1).

### Aims and objectives

1. The *primary aim* is to test the hypothesis that the rate of OS at 5 years with laparoscopic RH is not inferior to that of the abdominal RH.
2. The *secondary aims* are to assess the differences of progression-free survival (PFS), intraoperative and perioperative complications, surgical indicators, and life quality measurements.

### Recruitment and eligibility

Potential participants will be patients with newly diagnosed stage IB3 and IIA2 cervical cancer at the following hospitals: the Obstetrics and Gynecology Hospital of Fudan University, Zhongshan Hospital of Fudan University, Renji Hospital of Shanghai Jiao Tong University, Xinhua Hospital of Shanghai Jiao Tong University, Taizhou Hospital Affiliated Cancer Hospital of the University of Chinese Academy of Sciences, and First Affiliated Hospital of Wenzhou Medical University in China. The main center is at the Obstetrics and Gynecology Hospital of Fudan University, which has an annual surgical volume of around 800 stages IB3 and IIA2 cervical cancer cases, and the other centers each operates 80–200 cases per year. The sub-PIs are all experienced surgeons with proven qualifications. All these surgeons have participated in national competitions, and three surgeons have won the championship [14].

The study information is advertised at each center’s cervical cancer clinic. Potential participants will be approached and screened by dedicated research staff. Interested patients will be given more detailed information about the study background, randomization method, and relevant surgical procedures. The patient enrollment will be done by the chief attending physician. Priority will be given to enrolled patients when scheduling follow-up and relevant treatment.

#### Inclusion criteria

Patients should meet the following criteria:

1. Clinical diagnosis of stage IB3 and IIA2 squamous carcinoma, adenocarcinoma, or squamous adenocarcinoma of the cervix
2. 70 years ≥ age ≥ 21 years
3. Surgery type C of Q-M surgical staging
4. Normal liver (abnormal indicators of transaminase ≤ 3 U/L, the maximum value not exceeding 3 times the respective normal value) and kidney (creatinine < 50 mg/dL) functions and normal range of blood count (hemoglobin > 60 g/L, platelets > 70 × 10^9^/L, leukocytes > 3 × 10^9^/L)
5. No history of other types of malignant tumors
6. Not pregnant
7. Functional status: Karnofsky performance score ≥ 60
8. Agree to join the study and sign the informed consent form, compliant and cooperative with the follow-up procedures
9. No psychiatric disorders and other types of serious immune system disorders such as HIV infection, lupus erythematosus, and myasthenia gravis

#### Exclusion criteria

Patients meeting one of the following criteria will be excluded:

1. Patients with contraindications to various surgeries and are not suitable to undergo surgery
2. Patients who have received prior treatment for cervical cancer, including pelvic/abdominal radiotherapy irradiation, or neoadjuvant chemotherapy
3. Patients with recurrent cervical cancer
4. Patients with suspicious metastasis of pelvic lymph nodes with maximum diameter > 2 cm based on CT, MRI, or PET-CT (pelvic MRI + PET-CT or pelvic MRI + upper abdominal MRI/CT + chest CT)

### Randomization, blinding, and treatment allocation

All eligible patients should provide written informed consent before being randomized. This study uses a centralized block (block size = 6) randomization method to ensure equal number of patients (1:1) are randomly assigned to the laparoscopic/robotic RH vs. abdominal RH groups. An independent statistical team at the Clinical Trial Center of the Children’s Hospital of Fudan University performs the randomization scheme by creating and sequencing random seed numbers using the SAS 9.4 software. The random assignment sequence that determines the intervention protocols will be placed in sequentially numbered, non-transparent, sealed envelopes according to zone: the six subgroups for each zone will be placed sequentially in six small, non-transparent, sealed envelopes marked from 1 to 6, and then placed in the same large envelope marked with the corresponding zone number. The allocation sequence details will be kept from the investigators and surgeons. The central coordinator at the main center (Obstetrics and Gynecology Hospital of Fudan University) who is not involved in the implementation of the intervention or the outcome evaluation will administer the randomization assignment after the completion of baseline measurements. The enrollment is competitive, and the sub-center coordinator will contact the central coordinator before the inclusion of the first patient in each group. The central coordinator will follow the randomization scheme and assign the large envelopes with the group numbers to each center following the order of contact. Each sub-center will open the envelopes according to the order of signing informed consent form, and strictly follow the numbered order of the envelopes, and then inform the participating physicians of the assignment scheme and complete the written registration form. The surgeons will perform the operations according to the allocated random number. After obtaining pathological diagnosis, patients meeting the pathological exclusion criteria will be excluded.

Patients will be informed of the allocated intervention arm after completing the pre-surgery evaluation. Study members carrying out the surgery or patients will not be blinded. Research staff performing outcome measurements and data analyses will be blinded.

## Treatment/intervention

### Accreditation of participating surgeons

Surgeons skilled in cervical cancer surgery are selected from each participating hospital. To ensure study quality, the lead surgeon must have performed at least 50 surgeries each for both LRH and ARH with available medical records. To ensure the extent of surgical resection and surgical oncologic management of each surgeon, the surgical quality control team and the PIs will be on site to audit each surgeon’s operations and review unedited videos (1 ARH and 1 LRH) for quality measurement. All surgical procedures during the trial will be recorded and stored for documentation. The surgical quality control team will randomly select and review unedited videos, then give feedback to each center. If there are any deviations of the surgical procedure, the quality control team will communicate with the surgeon and provide a 1-month monitoring period for improvement and re-evaluation. Additionally, sensitive analysis will be performed during the data analysis phase.

### Surgery therapy

In this study, the surgeon can choose either laparoscopic radical cervical cancer treatment (LRH) or robotic-assisted laparoscopic radical cervical cancer treatment in the minimally invasive radical cervical cancer treatment arm. Since this study focuses on the survival benefits after minimally invasive or open radical cervical cancer treatment, radical cervical cancer treatment with preservation of reproductive function will be excluded. For both open and minimally invasive radical surgeries, first a thorough abdominal exploration is performed to carefully explore the diaphragm, and detail in the operative record any metastatic lesions and/or metastatic sites, which will be confirmed by biopsy. If intra-abdominal lesions are found, radical cervical cancer surgery should be abandoned and switch to palliative treatment.

During radical cervical cancer surgery, pelvic lymph node dissection should be performed. In this project, sentinel lymph node mapping (SLN) is not included due to insufficient evidence for its sensitivity and specificity. For those patients with stage IB3 and IIA2 cervical cancer tumor lumps (diameter > 4 cm), paraaortic lymph node dissection is preferred and followed by paraaortic lymph node biopsy. It is sufficient for the upper border to reach the inferior mesenteric artery when clearing paraaortic lymph nodes. The common iliac lymph nodes are sent separately for examination, with the resection including both sides of the common iliac vessels (upper edge reaching the midpoint of the bifurcation of the abdominal aorta and the common iliac vessels, and lower range reaching the bifurcation of the common iliac vessels).

In case of definite pelvic lymph node metastasis intraoperatively, pelvic lymph node dissection and/or radical cervical cancer surgery could be discontinued, but it is recommended to perform abdominal para-aortic lymph node sampling to assess the degree of disease progression and develop corresponding radiotherapy regimens. If decide to continue surgery, it is recommended to perform radical cervical cancer surgery + pelvic lymph node dissection + abdominal paraaortic lymph node dissection/biopsy.

The surgical approach for stage IB3 and IIA2 is type C2 (radical hysterectomy + bilateral pelvic lymph node dissection + paraaortic lymph node dissection, 3–4 cm of parametrium and upper 1/4 to 1/3 of the vagina) (surgical staging based on FIGO 2018 and surgical staging based on Q-M staging). Parametrial tissue should be removed to a depth below the deep uterine vein, and 1–2 cm of parametrial tissue should be removed when suspecting preoperative involvement of the vaginal wall. If the decision of ovarian preservation is made after evaluating the patient condition, age, and willingness, ovarian transposition must be performed during the operation to the lower edge of the kidney.

There will be no alterations to usual care pathways (including any medication usage) when implementing laparoscopic radical hysterectomy or abdominal hysterectomy, and will remain for both trial arms.

### Surgical oncologic principles

To reduce the risk of tumor recurrence, the following tumor-free principle details are specified, especially for the endoscopic surgery.

1. Severing the vagina after vaginal closure by: closure of the vagina with an obturator, ligature ring ligation of the vagina, transvaginal suture of the vagina.
2. After laparoscopic ligation of the vagina, the vagina is irrigated with sterile water, and then severed and sutured; after open surgery, the vagina is closed with renal clamps and the anterior vaginal wall is incised, gauze is inserted, then the posterior wall is severed, and the vaginal stump is sterilized.
3. Encouraging sharp and gentle excision and avoiding vigorous tearing and plucking movements.
4. Removing whole lymph nodes without touching other parts and placing in a specimen bag for final removal; performing extensive hysterectomy in whole and avoiding excessive straining intraoperatively.
5. Rinsing the instruments that have contacted the tumor in the vagina and separating them from other instruments; if suspicious metastatic tissue is encountered in the pelvic and abdominal cavity, directly removing and depositing it into the specimen bag without contacting other parts after excision.
6. Aspirating the pelvic and abdominal cavity after full postoperative rinsing with sterile water and avoiding backflow of pelvic rinsing fluid into the upper abdomen when the head is low and the feet are high.
7. Do not use intrauterine manipulators; instead, use other methods such as silk thread to pull the uterine body.

## Post-operative adjuvant radiotherapy and/or radiochemotherapy

Adjuvant treatments are standardized across all participating centers in the study. At the beginning of the study, a meeting including the Radiotherapy Departments of all participating centers was held to develop a standardized adjuvant treatment protocol based on the NCCN guidelines.

Extended-field radiation therapy (EFRT) was utilized in patient with para-aortic positive nodal disease. If pathology confirms positive abdominal aortic or positive common iliac lymph nodes, irradiation of para-aortic extended field will be performed. If the pelvic lymph nodes are positive and the pathology result of abdominal aortic lymph nodes clearance is negative (if only the main abdominal biopsy is performed without dissection, positive irradiation will be performed, and negative equivalent to no dissection), no extended field irradiation will be performed. If pelvic lymph nodes are positive and para-aortic lymph nodes not been dissected, but imaging result suspects para-aortic lymph node metastasis (full lymph nodes, length to diameter ratio close to 1), then extend field irradiation is recommended.

## Follow-up procedures

Follow-up visits will be performed at the dedicated unit at each participating center. Written informed consent should be given before starting any protocol-related procedures. Table 1 presents detailed study procedures and assessments at baseline and/or follow-up [13, 14].

**Table 1:** Study procedures and assessments at baseline and follow-up

Visit no	Eligibility screening	Randomization	Blood examination, urinary test, liver and renal function, ECG	Surgical indicators, surgical complications	Gynecological examination	Imaging test	SCCA/CA-125	Cervical liquid-based cytology	HPV test	Adverse events	EORTC QLQ-C30 v3.0; EORTC QLQ-CX24
V0	×										
V1		×	×	×	×	×	×	×	×	×	
V2 (3 months)					×	×	×	×		×	×
V3 (6 months)					×	×	×	×	×	×	×
V4 (9 months)					×	×	×	×		×	×
V5 (1 year)					×	×	×	×	×	×	×
V6 (15 months)					×	×	×	×		×	×
V7 (1.5 years)					×	×	×	×		×	×
V8 (21 months)					×	×	×	×		×	×
V9 (2 years)					×	×	×	×		×	×
V10 (2.5 years)					×	×	×	×	×	×	×
V11 (3 years)					×	×	×	×	×	×	×
V12 (3.5 years)					×	×	×	×	×	×	×
V13 (4 years)					×	×	×	×	×	×	×
V14 (4.5 years)					×	×	×	×	×	×	×
V15 (5 years)					×	×	×	×	×	×	×

*ECG* electrocardiogram, *EORTC* European Organization for Research and Treatment of Cancer, *QLQ* Quality of Life Questionnaire, *SCCA* SCC antigen test

To promote participant retention and complete follow-up, study team members at each center will give detailed patient orientation during the recruitment phase. The study team members will provide remote condition assessment services and consultation services for enrolled patients during the study period. The follow-up system will remind each patient her up-coming follow-up visit and related items. To reduce the loss to follow-up rate, dedicated research staff will perform the regular follow-up. In case of patient withdrawal, the investigator will make efforts to assess and document the reasons and obtain the patient’s consent to continue monitoring the disease status (relapse, survival, toxicity, etc.) through the patient’s medical record. If a patient moves to another study center and changes physician, the investigator will seek to locate that center’s physician and request assistance in order to complete the follow-up.

## Outcome measurements

### Primary outcome measurements

The primary outcome will be 5-year overall survival (OS) rate.

### Secondary outcome measurements

Secondary outcomes include 5-year PFS; operation time, anesthesia time, intraoperative bleeding volume, intraoperative complications, postoperative complications, postoperative pain score, postoperative hospital stay, 1-month postoperative survival quality, 1-year post operative survival quality, and sexual quality of life.

## Description of statistical methods

### Sample size calculation

This study is a non-inferiority trial, with the primary outcome of 5-year OS rate. The sample size is calculated based on the difference between the two groups of 5-year OS rate. According to clinical data from our hospital, the 5-year OS rate of the open surgery group is estimated to be 72%. Based on the assumptions of (i) the 5-year OS rate of 72% in the open surgery group, (ii) a non-inferiority margin of 8%, (iii) 80% power, and (iv) a one-sided alpha of 0.025, the sample size estimation resulted in 495 subjects per group (SAS software, version 9.4). Assuming a 10% drop-out rate and considering the randomization scheme with a block size of 6, the final sample size is 1104 patients (552 for each group).

### Planned analyses

Intention-to-treat (ITT) analysis will be performed using the full analysis set, which includes every patient who is randomized based on treatment assigned. Missing data will be censored at the last date the patient is known to be alive. Sensitivity analysis will be performed using the per-protocol (PP) population, which includes those patients who complete the treatment without any major protocol violations. There is one interim analysis planed using the O’Brien Fleming method, which will occur after half of enrolled patients are followed for 2 years. All analyses will be performed using the SAS software, version 9.4 (SAS Institute), and a two-sided significance level of 0.05 will be considered as statistically different.

- 1. Analysis of primary outcome data
  The curves of OS at 5 years will be estimated using the Kaplan–Meier method, and compared by the log-rank test. The difference of the 5-year OS rate with its 95% confidence interval (CI) between the two groups will be estimated. If the one-sided 95% upper limit is less than, where the predetermined non-inferiority margin = 8%, then the minimally invasive surgery group will be considered non-inferior to the open surgery group. Cox proportional hazards regression model will be used to obtain the hazard ratio and corresponding 95% CI, adjusting variables such as blood loss during operation, operative duration, and postoperative pain score. In addition, stratified analysis will be performed according to tumor stage.
- 2. Analysis of secondary outcome data
  Variables including operative duration, blood loss during operation, anesthesia time, postoperative pain score, and postoperative hospital stay are considered as continuous outcomes. Continuous outcomes with normal distribution will be presented as mean ± standard deviation (SD), while outcomes not following normal distribution will be presented as median (interquartile). The differences of the outcomes and corresponding 95% CIs will be analyzed using generalized linear model (GLM) with treatment as fixed effect, assuming normal distribution and using identity link function.

Categorical variables including intraoperative complications, postoperative complications, 1-month and 1-year postoperative quality of life, and sexual life will be summarized using number and percentage of patients with the event. The differences of the outcomes and corresponding 95% CIs will be analyzed using GLM with treatment as fixed effect, assuming binomial distribution and using identity link function.

Adverse events (AEs) will be reported using the number of AEs, the number and percentage of participants with AEs by each group. The rate of complications will be closely monitored and assessed 3 months postoperatively after 50, 100, 150, 200, and 300 patients are randomized into each group.

## Patient and public involvement statement

There is no patient or the public involvement in the study design, conduct, reporting, or dissemination plans of this research. Manuscripts of the study results will be prepared and submitted for publication in accredited international medical journals and presented at academic conferences. Study results will also be communicated via participating hospital’s websites.

## Data confidentiality and storage

After patients’ consent, the research assistant will collect patient personal information, medical history from medical records. Data will then be quality checked and double entered into the study electronic database. Paper copy data will be stored in a filing cabinet with lock, and electronic data will be stored on a password-protected computer at a designated research office in the hospital, which only can be accessed by research staff. Follow-up data will be linked to the baseline clinicopathological database via unique patient ID number. All identifiable information will be removed before data analysis.

## Ethics and dissemination

This study was approved by the Institutional Review Board of the Obstetrics and Gynecology Hospital of Fudan University in Shanghai, China (Reference number: 2021–05; date of approval: 18 January 2021), and is conducted in accordance with the Declaration of Helsinki.

All patients will be given both written and oral information about the study from their chief attending gynecologist during admission for surgery. Patients must sign an informed consent form in accordance with the Declaration of Helsinki before being included in the study. Patients refusing to participate will then receive standard treatment. Patients can withdraw from the study without any reason at any time during the study period. The patients or their family members can address their concerns or queries to the project leaders (HJ or XW). Investigators have the right to withdraw a patient from the trial intervention or study in the event of secondary diseases, adverse events, protocol violations, and administrative or other reasons. There is no anticipated harm and compensation for trial participation; therefore, no provision for post-trial care is planned.

## Discussion

Cervical cancer remains one of the main gynecologic malignancies in developing countries [17]. In China, both the incidence and mortality of cervical cancer has shown an increasing trend. In 2018, there were nearly 110,000 new cases and nearly 50,000 deaths of cervical cancer in China, accounting for approximately 20% and 16% of the global new cases and deaths, respectively [1]. HPV vaccine has limited effect for those already infected [18]. In addition, the HPV vaccination program has only been recently implemented in China [19]. Therefore, cervical cancer is and will remain to be an important health problem in China [17], and it is crucial to choose both clinically and culturally acceptable as well as cost-effective treatment strategies.

A retrospective study in Europe in 2020 [20] showed that for minimally invasive radical laparoscopic, if tumor-free techniques were performed (such as not using intrauterine manipulator, and clipping the vagina at the designated location and opening the vagina wall below), then minimally invasive radical laparoscopic is equivalent to abdominal radical hysterectomy in terms of PFS and recurrence rate. During the past 8 years, our hospital has an annual surgical volume of about 1500–2000 cervical cancer cases. Our data showed that the 5-year OS rate is 100% and 96.93% for stage IA2 and stage IB1 cervical cancer patients respectively, similar to laparotomy in the LACC trial [10]. For IB3 and IIA2 patients, it is more challenging to totally remove the larger tumor and strictly follow the surgical oncologic procedures. Therefore, the current study set stringent criteria in terms of the qualification of participating surgeons and corresponding surgical oncologic procedures.

The debate of laparoscopic versus abdominal RH is still ongoing, and high-quality evidence is needed to guide clinical practice, as well as improve specific techniques such as for minimally invasive surgery. In this randomized trial, we will follow strict surgical oncologic procedures and avoid using uterine manipulator during endoscopy. We aim to evaluate the efficacy of the two surgeries, comparing the survivals of patients receiving minimally invasive radical hysterectomy versus abdominal radical hysterectomy for stages IB3 and IIA2 cervical cancer patients. In addition, we plan to evaluate the short-term and long-term quality of life of these patients. The study results will provide more convincing evidence-based information for stages IB3 and IIA2 cervical cancer patients and their gynecologic cancer surgeons in their choice of surgical method.

## Trial status

The study was approved by the Institutional Review Board of the Obstetrics and Gynecology Hospital of Fudan University in Shanghai, China, on 18 January 2021. The trial was retrospectively registered on the ClinicalTrials.gov, and the registration number was obtained on 25 June 2021. Recruitment of participants started in May 2021 and is estimated to finish in May 2024. The last participant is expected to reach the primary endpoint (5-year follow-up) in May 2029. Primary data analysis will begin in May 2026. The protocol version number and date were 1.0 and 21 December 2020, respectively.

### Supplementary Information

**Additional file 1.**

**Additional file 2.**

**Additional file 3.**

## Data Availability

The full protocol could be downloaded at ClinicalTrials.gov (NCT04939831, https://www.clinicaltrials.gov/ct2/show/NCT04939831?term=NCT04939831&draw=2&rank=1). Data generated during this study and statistical code are available upon reasonable request from the study PIs after approval by the trial steering committee.

## Abbreviations

CCRT: Concurrent chemoradiotherapy
DFS: Disease-free survival
DMC: Data monitoring committee
ECG: Electrocardiogram
EORTC: European Organization for Research and Treatment of Cancer
FIGO: International Federation of Gynecology and Obstetrics
HPV: Human papillomavirus
HR: Hazard ratio
ITT: Intention-to-treat
LVSI: Lymphovascular space invasion
NCCN: National Comprehensive Cancer Network
OS: Overall survival
PFS: Progression-free survival
PROBE: Prospective, randomized, open, blinded endpoint
PP: Per protocol
QLQ: Quality of Life Questionnaire
Q-M: Querleu-Morrow
RCT: Randomized clinical trial
RH: Radical hysterectomy
SCCA: SCC antigen test
95%CI: 95% Confidence interval

## References

[1] Bray F, Ferlay J, Soerjomataram I, Siegel RL, Torre LA, Jemal A (2018). Global cancer statistics 2018: GLOBOCAN estimates of incidence and mortality worldwide for 36 cancers in 185 countries. CA Cancer J Clin.

[2] Cibula D, Pötter R, Planchamp F (2018). The European Society of Gynaecological Oncology/European Society for Radiotherapy and Oncology/European Society of Pathology Guidelines for the Management of Patients With Cervical Cancer. Int J Gynecol Cancer.

[3] Aarts JWM, Nieboer TE, Johnson N, Tavender E, Garry R, Mol BW, Kluivers KB (2015). Surgical approach to hysterectomy for benign gynaecological disease. Cochrane Database Syst Rev.

[4] Magrina JF, Kho RM, Weaver AL, Montero RP, Magtibay PM (2008). Robotic radical hysterectomy: comparison with laparoscopy and laparotomy. Gynecol Oncol.

[5] Geisler JP, Orr CJ, Khurshid N, Phibbs G, Manahan KJ (2010). Robotically assisted laparoscopic radical hysterectomy compared with open radical hysterectomy. Int J Gynecol Cancer.

[6] Roque DR, Wysham WZ, Soper JT (2014). The surgical management of cervical cancer: an overview and literature review. Obstet Gynecol Surv.

[7] Nam JH, Park JY, Kim DY, Kim JH, Kim YM, Kim YT (2012). Laparoscopic versus open radical hysterectomy in early-stage cervical cancer: long-term survival outcomes in a matched cohort study. Ann Oncol.

[8] Bogani G, Cromi A, Uccella S, Serati M, Casarin J, Pinelli C, Ghezzi F (2014). Laparoscopic versus open abdominal management of cervical cancer: long-term results from a propensity-matched analysis. J Minim Invasive Gynecol.

[9] Melamed A, Margul DJ, Chen L (2018). Survival after minimally invasive radical hysterectomy for early-stage cervical cancer. N Engl J Med.

[10] Ramirez PT, Frumovitz M, Pareja R (2018). Minimally invasive versus abdominal radical hysterectomy for cervical cancer. N Engl J Med.

[11] Landoni F, Maneo A, Colombo A, Placa F, Milani R, Perego P, Favini G, Ferri L, Mangioni C (1997). Randomised study of radical surgery versus radiotherapy for stage Ib-IIa cervical cancer. Lancet.

[12] Wang W, Hao M, Chen CL (2019). Trend in proportion and clinicopathological characteristics of young women with stage Ia2 to IIa2 cervical cancer. Zhonghua Fu Chan Ke Za Zhi.

[13] Wu X, Feng H, Gao S (2022). A multicenter noninferior randomized controlled study comparing the efficacy of laparoscopic versus abdominal radical hysterectomy for cervical cancer (stage IA1 with LVSI, IA2): study protocol of the LAUNCH 1 trial. BMC Cancer.

[14] Wu X, Qiu L, Lou W, et al. A multicenter non-inferior randomized controlled study comparing the efficacy of laparoscopic versus abdominal radical hysterectomy for cervical cancer (stages IB1, IB2, and IIA1): study protocol of the LAUNCH 2 trial. 2022;23(1):269. 10.1186/s13063-022-06245-5. 10.1186/s13063-022-06245-5 PMC8991786 35395868

[15] Chan AW, Tetzlaff JM, Altman DG (2013). SPIRIT 2013 statement: defining standard protocol items for clinical trials. Ann Intern Med.

[16] Calvert M, Kyte D, Mercieca-Bebber R (2018). Guidelines for inclusion of patient-reported outcomes in clinical trial protocols: the SPIRIT-PRO extension. JAMA.

[17] Chen W, Zheng R, Baade PD, Zhang S, Zeng H, Bray F, Jemal A, Yu XQ, He J (2016). Cancer statistics in China, 2015. CA Cancer J Clin.

[18] Wang R, Pan W, Jin L, Huang W, Li Y, Wu D, Gao C, Ma D, Liao S (2020). Human papillomavirus vaccine against cervical cancer: opportunity and challenge. Cancer Lett.

[19] Zou Z, Fairley CK, Ong JJ, Hocking J, Canfell K, Ma X, Chow EPF, Xu X, Zhang L, Zhuang G (2020). Domestic HPV vaccine price and economic returns for cervical cancer prevention in China: a cost-effectiveness analysis. Lancet Glob Health.

[20] Chiva L, Zanagnolo V, Querleu D (2020). SUCCOR study: an international European cohort observational study comparing minimally invasive surgery versus open abdominal radical hysterectomy in patients with stage IB1 cervical cancer. Int J Gynecol Cancer.

