# Supplementary material for: A multicenter noninferior randomized controlled study comparing the efficacy of laparoscopic versus abdominal radical hysterectomy for cervical cancer (stage IB3 and IIA2): study protocol of the LAUNCH 3 trial
Source: Trials. 2023 Aug 18;24:542. doi: 10.1186/s13063-023-07573-w (PMC10436641; doi:10.1186/s13063-023-07573-w)
Supplement: Supplementary file 2 — Additional file 2. [file 13063_2023_7573_MOESM2_ESM.docx]

You will be invited to participate in a clinical research study. These instructions give you some information to help you decide whether to participate in this clinical study. Please read it carefully and ask the investigator in charge of the study if you have any questions. Your participation in this study is voluntary. This study has been reviewed by our Institutional Ethics Committee.

**Why is this study being conducted? What is the purpose of this study?**

We are conducting a study in which subjects with early-stage cervical cancer are randomized to compare the postoperative outcomes of subjects who undergo radical laparoscopy (both conventional and robot-assisted laparoscopy) with those who undergo open radical laparoscopy. Cervical cancer is the most common malignancy of the female reproductive system in the worldwide. Surgery is the main treatment for early-stage cervical cancer (including IA1, IA2, IB1, IB2, IIA1) and can also be used for the initial treatment of stages IB3 and IIA2. However, the advantages and disadvantages of the two surgical approaches are controversial, and it is not clear which one has a better prognosis for cervical cancer subjects (data from an international randomized controlled clinical study suggest that open is superior to laparoscopy, while previous data from our institution and some Chinese data suggest that laparoscopy has similar survival rates to open). Therefore, we wanted to draw our conclusions using a multicenter randomized controlled clinical study in China.

Primary objective: To compare oncologic outcomes in subjects with early-stage cervical cancer who undergo laparoscopic radical cervical cancer surgery with those who undergo conventional open radical cervical cancer surgery.

Secondary objective: To observe the differences in operative time, anesthesia time, intraoperative bleeding, intraoperative complications, postoperative recovery time, postoperative complications, length of hospital stay, quality of survival at one month postoperatively, quality of survival and sexual quality of life assessment at one year postoperatively, and cost of surgery in subjects with early-stage cervical cancer who undergo minimally invasive and open radical cervical cancer surgery. In addition, in order to investigate the mechanism of cervical cancer and guide individualized targeted and immunotherapy, we may need to collect your tumor tissues and normal tissues adjacent to the cancer, metastases, serum, etc. for genetic testing, protein levels and other related studies.

**Who is conducting the study? Where is the study being conducted?**

The study will be conducted simultaneously at the Obstetrics and Gynecology Hospital of Fudan University, Zhongshan Hospital of Fudan University, Taizhou Cancer Hospital (Taizhou Campus of Zhejiang Cancer Hospital), the First Hospital of Wenzhou Medical University, Xinhua Hospital of Shanghai Jiao Tong University School of Medicine, and Renji Hospital of Shanghai Jiao Tong University School of Medicine, and is expected to enroll patients for 3 years. The study protocol will be sent to the appropriate hospital ethics committee of each participating team for prior review and approval. Written informed consent will be obtained from all subjects. The relevant surgical procedures will be performed on the subjects by clinicians from the corresponding medical teams of the relevant hospitals who meet the requirements for surgical operation, and the subjects or their families will be followed up by the relevant study team members for a period of 5 years. There are no external investigators or other investigators.

**Who will be invited to conduct this study? How many people will be enrolled in this study?**

Subjects recruited will meet several of the following criteria:

a) Patients with a clinical diagnosis of stage IB3, IIA2 cervical cancer.

b) Preoperative pathological diagnosis of squamous carcinoma of the cervix, adenosquamous carcinoma of the cervix or adenocarcinoma of the cervix.

c) 21 years ≤ age ≤ 70 years.

d) Normal liver and kidney function.

e) No history of other malignancies.

f) Non-pregnant.

g) Subjects are able to tolerate surgery.

h) Physical fitness rating: Karnofsky score ≥ 60.

i) First-time treatment recipient.

j) Subjects who have good compliance are willingly to enroll in this study, sign informed consent, and cooperate with follow-up.

k) No psychiatric disorders and other serious immune system diseases (e.g. SLE, myasthenia gravis, HIV infection, etc.).

(Note: Maximum diameter measurement of cervical lesions is based on MRI or CT or PET-CT)

We will recruit 690 eligible subjects from October 2020 to December 2023 (total 3 years) at the Obstetrics and Gynecology Hospital of Fudan University, Zhongshan Hospital of Fudan University, Xinhua Hospital of Shanghai Jiao Tong University School of Medicine, Renji Hospital of Shanghai Jiao Tong University School of Medicine, Cancer Hospital of the Chinese Academy of Sciences University, and the First Affiliated Hospital of Wenzhou Medical University. The subjects will be randomized 1:1 and undergo either conventional laparoscopic surgery or open laparotomy. Enrollment will be completed from October 2020 to December 2023 with a 5-year follow-up. The follow-up endpoint is that the data analysis can be completed efficiently or this trial is terminated early by the ethics committee or independent monitoring institutions.

**What will I need to do if I participate in this study?**

Subjects participating in the study will come from the Obstetrics and Gynecology Hospital of Fudan University, Zhongshan Hospital of Fudan University, Zhejiang Cancer Hospital, the First Affiliated Hospital of Wenzhou Medical University, Xinhua Hospital of Shanghai Jiao Tong University School of Medicine, and Renji Hospital of Shanghai Jiao Tong University School of Medicine. If you volunteer to participate in this study, you will be randomized by the investigators to undergo either laparoscopic radical surgery (or robotic-assisted) or open radical surgery. Prior to surgery, you will be informed of the purpose of the study, the procedure to be performed, the risks associated with the procedure, postoperative complications, and postoperative follow-up, then you will sign a subject informed consent form.

Subjects will be randomly divided into two groups. Half of the subjects will be in the "minimally invasive surgery group" and the other half will be in the "open surgery group". A uniform strategy of radiotherapy or concurrent radiotherapy with or without chemotherapy will be used after surgery for subjects with risk factors for recurrence (see the 2020 NCCN guidelines for treatment criteria). Subjects may not change grouping, and such grouping is necessary so that the prognosis of the two surgical groups can be compared.

Before treatment, we will observe and record your basic information, all physical clinical tests, imaging indicators, genetic tests and other important data (including blood and urine routine, blood liver and kidney function, ECG, squamous cancer: SCC antigen test (SCCA)/adenocarcinoma: CA-125, HPV typing test, etc.). The end of the surgery does not mean the end of the study. You must complete the treatment according to the protocol given by your doctor and come to the hospital or local visits according to the follow-up schedule agreed between your doctor and you, and you need to keep close contact with the treatment team to inform the recent status. You will have the same follow-up program and frequency as regular patients outside the trial, but you will have the benefit of priority and free access to the treatment team for advice. You will also be required to upload relapse reports regularly and to fill in the scales provided by us at various times after treatment so that the treatment team can obtain accurate data on your recovery and social support. Our follow-up visits are based on the delivery of reports by WeChat, supplemented by telephone follow-up visits. Your follow-up data will be properly and meticulously recorded and studied as a whole together with other patients' data. The content of the follow-up: review every 3 months for 2 years after completion of treatment including imaging (pelvic enhancement MRI or chest CT + pelvic enhancement MRI + upper abdomen enhancement MRI or PET-CT + pelvic enhancement MRI); SCC antigen testing (SCCA) and CA-125; vaginal stump fluid-based cytology and HPV testing (once a year after completion of treatment); record of adverse events and serious adverse events. Reviewed every 6 months after 2 years until the end of the study, including: gynecological examination, SCC antigen test (SCCA), CA-125, vaginal stump fluid-based cytology and HPV, imaging (pelvic enhancement MRI or chest CT + pelvic enhancement MRI + upper abdomen enhancement MRI or PET-CT + pelvic enhancement MRI), and adverse events will be recorded until the end of the study.

**What are the risks associated with this study? What problems will I face by participating in this study?**

All gynecological tumor surgeries may have complications such as intraoperative and postoperative bleeding, cardiovascular and cerebrovascular accidents, and peripheral organ damage (which will be answered by your surgeon), and chemotherapy or simultaneous radiotherapy may have adverse effects such as bone marrow suppression, gastrointestinal reactions, neurological damage, and radiotherapy may have radiological inflammation of peripheral organs, which are also adverse effects that occur in conventional treatment. We will give appropriate treatment according to the relevant regulations.

**What are the benefits of this study?**

Subjects enrolled in this study will receive priority treatment at our hospital and will be operated on by the lead surgeon of an excellent surgical team with extensive surgical experience. You will also be followed up after surgery by a dedicated person. Your 5-year survival rate will likely be improved, your post-operative complications will likely be reduced, your hospital stay will likely be shortened, and your quality of life will likely be further improved. Finally, the results of this study will have implications for future trends in gynecologic oncology surgery in China and around the world, bringing more suitable surgical options to more subjects.

**Is there a fee for this study? Will I receive any compensation?**

There will be no additional charges for this study other than the normal consultation fees, and we will not pay any fees for your participation. The lead surgeon of each surgical team will be in charge during the surgical phase, free online consultation reports during the trial phase, and priority access to treatment when problems arise are the benefits that subjects receive. If the subject suffers complications related to the medical procedure, follow-up medical care will be provided by this medical institution, and the responsibility will be determined by the relevant institution, with the corresponding compensation as for the regular medical procedure.

**What are my options if I do not want to participate in this study?**

If you choose not to participate in the study, you will be seen by your doctor according to the usual gynecologic oncology protocols. Whether you participate in the study or not will not affect your relationship with your doctor, nor will there be any loss of medical or other benefits to you.

**What are my rights as a participant in the study?**

Participation in this study is totally voluntary and you do not have to feel pressured to participate. If you do not want to participate in this study, it will not affect your hospital care or treatment in any way. You may withdraw from the study at any time. In the meantime, we will provide subjects with literature related to this study.

**Is there anything else I should know?**

Subjects and family members participating in this study may be contacted by a member of the study team to answer questions. At that time, you will be asked if you would like to participate in the study. Although you currently meet the enrollment criteria and are fully volunteering for this study, it does not mean that you will ultimately be included in the study. If there is any important new information during the course of the study that may affect your willingness to continue to participate in the study, you will be notified promptly and you may ask any questions about this study at any time and have them answered accordingly.

**Under what circumstances will I be informed that my continued participation in the study will be terminated?**

In your best interest, your doctor or investigator may discontinue your continued participation in this study at any time during the study if

- You are found to have a contraindication to the procedure in question during the study and cannot undergo the procedure.

- The study is terminated or cancelled at the request of the sponsor.

- You have received prior pelvic/abdominal radiation exposure or neoadjuvant chemotherapy for cervical cancer.

- You have a CT, MRI or PET report indicating that your lesion has metastasized outside the cervix.

- You are unable or unwilling to sign an informed consent form and comply with study requirements.

**Other information that may be helpful to you**

As a national clinical class I key specialty and a key discipline of the Ministry of Education, the Obstetrics and Gynecology Hospital of Fudan University undertakes the important task of diagnosis and treatment of malignant tumors of the female reproductive system and scientific research nationwide, and has the second to none experience in diagnosis and treatment of gynecological malignant tumors and scientific research base in China, treating about 1500-2000 new cases of cervical cancer every year, accounting for about 50% of the cases in Shanghai. Our doctors have rich clinical experience and can provide you with the most suitable treatment support.

We would be interested in hearing from you and answering your questions, including any questions about this study. For more information or if you have any suggestions, questions or concerns about this study, please contact [sky_xin1980@aliyun.com](mailto:sky_xin1980@aliyun.com) by email or call (021)33189900-6529 from 7a.m. to 8a.m.

**Who will see, use or analyze your personal information? and your privacy?**

A copy of such an informed consent form will be included in your medical record materials.

If the treatment you receive at our hospital is related to your condition, the medical information collected in this study will be part of your case record at the hospital and the doctor will record the results of the laboratory tests in your medical record. Your medical records (study chart/CRF, laboratory reports, etc.) will be kept intact in our medical history room. Any public reports of the results of this study will not disclose your personal identity. Every effort will be made to protect the privacy of your personal medical information to the extent permitted by law. In general, anyone involved in this study, including ethics committees and drug regulatory authorities may see data, including your personal information. For instance, the following people may see your personal information.

- Researchers at the Obstetrics and Gynecology Hospital of Fudan University who are involved in this study.

- Medical staffs at the Obstetrics and Gynecology Hospital of Fudan University who are directly involved in the treatment and care associated with the study.

- Researchers from other research centers involved in the study, including those supervising the study at the center.

- Personnel at the Obstetrics and Gynecology Hospital of Fudan University who were responsible for overseeing, advising and evaluating the study and care, including the hospital ethics committee.

- Those responsible for overseeing study information, such as data security monitoring committees, clinical research organizations, data centers, statisticians for the study, including government or related companies that manufacture the drugs or devices used in this study.

Your information and treatment data will be stored in our study center and will also be aggregated at the Shanghai Shenkang Hospital Management Center, and if certain government policies require us to share information, we will follow the regulations and upload it to the designated platform. We will make every effort to protect the privacy of your personal medical information to the extent permitted by law.

We will create a number for the research information we collect about you so that identification of the information will be kept separate from the data. The results of this research may be used for publication in medical-related journals or books, or for teaching purposes. However, our use will be limited to the research data and results, and your name or personally identifiable information will not be used.

**Contact Information**

I understand that if you have any questions or concerns about this study, you may contact the study person/office using the contact information below.

| 🚹 Person to contact | **🕾** Contact Information | | **❓** When I have any questions or concerns |
| --- | --- | --- | --- |
| Research Contact: | Phone: | 021-33189900-6529 | ▪General research-related questions  ▪Handling of possible injuries or emergencies that may arise from the study  ▪Any research-related questions or complaints |
| Dr. Xin Wu |  |  |  |
| Research team members：  Dr.Hao Feng, Ling Qiu, and Hailin Yu | Phone: | 021-33189900-6529 | ▪General research-related questions  ▪Handling of possible injuries or emergencies that may arise from the study  ▪Any research-related questions or complaints |
|  | Main contact information | Through the Wechat clinical follow-up platform |  |
| Ethnics committee office： | Phone: | 021-33189900 | ▪ Rights if study subjects  ▪ Use of protected research-related information  ▪ Research-related costs or harm  ▪ Any research-related questions or complaints  ▪ Any questions that the researchers and research team members cannot answer  ▪ If you wish to communicate with someone other than the researcher or research team members only |
|  |  |  |  |

**Informed Consent and Authorization Document**

▪ I have carefully read the informed consent form associated with this study and have been given sufficient time to consider or discuss with my family whether to participate in this study.

▪ The researcher has explained the study to me in detail, including the possible risks and benefits, which are of concern to me.

▪ All my questions have been answered and responded to satisfactorily.

▪ I understand that participation in this study is completely voluntary and that I may withdraw from the study at any time.

▪ This informed consent is signed by me before I participate in any of the studies.

▪ I consent to my/my family's participation in this study and to the use of the protected personal data described above.

◼ __________________ ____________________________________________ _________________

Date (yyyy/mm/dd) Subject's signature Relationship to subject

**Authorization to sign informed consent form**

I consent to the signing of documents related to the study by ________ as my authorized person to sign and to the use of my protected personal information and other information.

◼ __________________ ____________________________________________ _________________

Date (yyyy/mm/dd) Authorized person's signature Relationship to subject

◼ __________________ ____________________________________________ _________________

Date (yyyy/mm/dd) Signature of the authorized person Relationship to subject

**Declaration and signature of the investigator or partner**

▪ I have fully explained the above research project, including the possible risks and benefits, to all interested parties, including the subjects and their families or authorized persons.

▪ I have explained and will explain to the best of my ability all inquiries related to this research project.

▪ I will notify all relevant authorities of any changes in study procedures or risks and benefits during or after the study has been explained

▪ I have provided an informed consent form signed by the subject/family of the subject/authorized person of the subject.

◼ __________________ ____________________________________________

Date (yyyy/mm/dd) Signature of Study Director

▪Signature is required only in the event of:

▪ □Informed consent must be read to the subject or her family or authorized person or

▪ □The subject's ability to give consent is limited by a communication or comprehension disorder, or

▪ □Other circumstances: Please specify _____________________________________________

▪ I confirm that the information in this consent form has been accurately explained and answered to the subject and his/her family or authorized person as required, and that the subject has given informed consent and voluntarily provided personal information related to the study.

◼ __________________ ____________________________________________

Date (yyyy/mm/dd) Signature of third party witness
