# Supplementary material for: A multicenter noninferior randomized controlled study comparing the efficacy of laparoscopic versus abdominal radical hysterectomy for cervical cancer (stage IB3 and IIA2): study protocol of the LAUNCH 3 trial
Source: Trials. 2023 Aug 18;24:542. doi: 10.1186/s13063-023-07573-w (PMC10436641; doi:10.1186/s13063-023-07573-w)
Supplement: Supplementary file 3 — Additional file 3. [file 13063_2023_7573_MOESM3_ESM.pdf]

A multicenter noninferior randomized controlled study comparing the efficacy of laparoscopic versus abdominal radical hysterectomy for cervical cancer

(Stage IB3, IIA2)

(Protocol number: NCT04939831)

Principle investigator: Hua Jiang (Obstetrics and Gynecology Hospital of Fudan University) (signed, dated December 2020)

Project team members: Xin Wu, Weili Yan (Children's Hospital of Fudan University), Xipeng Wang (Xinhua Hospital), Libing Xiang (Zhongshan Hospital), Weihua Lou (Renji Hospital), Tao Zhu (Taizhou Hospital, Affiliated Cancer Hospital of the University of Chinese Academy of Sciences), Yuyang Zhang (The First Affiliated Hospital of Wenzhou Medical University), Yongrui Bai (Renji Hospital), Yanli Hou (Renji Hospital), Yan Du, Weiguo Hu, Zhiling Zhu, Xiaohong Xue, Hailin Yu, Hao Feng, Ling Qiu, Shurong Zhu (all people didn't noted above belong to Obstetrics and Gynecology Hospital of Fudan University)

OB&GY of Fudan University

Version number: 1.0, version date: December 21, 2020

Contents

I. Research Information
II. Research Background
2.1 Research Background
2.2 Research Objectives
2.3 Research Design
III. Research Methods
3.1 Research Setting
3.2 Eligibility Criteria
3.3 Interventions
IV. Allocation, Blinding and Unblinding
4.1 Method of Allocation of the Intervention
4.2 Blinding
V. Methods of Data Collection, Management and Analysis
5.1 Data Collection and Patient Retention
5.2 Data Management (Data entry, coding, encryption and storage schemes)
5.3 Statistical Analyses
VI. Quality of Life Questionnaire
6.1 Patient-reported Clinical Outcome Assessment Scale PRO 147 (V 1.0) for Cervical Cancer
6.2 Generic quality of life questionnaire
VII. Methods of Data Monitoring
7.1 Study Monitoring
7.2 Interim Analysis
7.3 Quality Control
VIII. Definition and Treatment of Adverse Events
8.1 Definition of Adverse Events
8.2 Observation, Recording and Treatment of Adverse Events
IX. Cost-benefit Analysis
X. Review
XI. Ethics and Dissemination
11.1 Ethical Approval
11.2 Study Protocol Modification Plan
11.3 Informed Consent
XII. Confidentiality
XIII. Declaration of Competing Interests
XIV. Data Collection
XV. Affiliation and Post-test Care
XVI. Dissemination Policy
XVII. Appendices

OB&GY of Fudan University

I. Research Information

1) Article title: A multicenter randomized, controlled, non-inferiority trial comparing the efficacy of laparoscopic versus abdominal radical hysterectomy for cervical cancer (Stage IB3and IIA2)

2) Clinical Trial Registration: the protocol has been registered by the Obstetrics and Gynecology Hospital of Fudan University at https://clinicaltrials.gov/, registration number: NCT04939831; all data sheets are included in the WHO clinical trial registration data (www.annals.org)

3) Version number: LAUNCH 3 1.0, version date: December 21, 2020.

4) Funding: Shanghai Shenkang Hospital Development Center's Shenkang Promotion of Clinical Skills and Clinical Innovation in Municipal Hospitals Three-Year Action Plan (2020-2023) Major Clinical Research Project (Grant No. SHDC2020CR1048B).

5) List of investigators and their **respective research roles and responsibilities**

Name	Affiliated Institution	Role
Hua Jiang	Obstetrics and Gynecology Hospital of Fudan University	Principle Investigator
Xin Wu	Obstetrics and Gynecology Hospital of Fudan University	Leader of Main Center
Xipeng Wang	Xinhua Hospital Affiliated to Shanghai Jiao Tong University School of Medicine	Leader of Sub-center
Weihua Lou	Shanghai Jiao Tong University School of Medicine, Renji Hospital	Leader of Sub-center
Libing Xiang	Zhongshan Hospital Affiliated to Fudan University	Leader of Sub-center
Tao Zhu	Taizhou Hospital Affiliated Cancer Hospital of the University of Chinese Academy of Sciences	Leader of Sub-center
Yuyang Zhang	The First Affiliated Hospital of Wenzhou Medical University	Leader of Sub-center
Weiguo Hu	Obstetrics and Gynecology Hospital of Fudan University	Clinical Researchers
Zhiling Zhu	Obstetrics and Gynecology Hospital of Fudan University	Clinical Researchers
XiaohongXue	Obstetrics and Gynecology Hospital of Fudan University	Clinical Researchers
Yongrui Bai	Shanghai Jiao Tong University School of Medicine, Renji Hospital	Radiotherapy researcher
Yanli Hou	Shanghai Jiao Tong University School of Medicine, Renji Hospital	Radiotherapy researcher
Weili Yan	Children's Hospital of Fudan University	Statistician

OB&GY of Fudan University

Yan Du	Obstetrics and Gynecology Hospital of Fudan University	Epidemiologist
Shujun Gao	Obstetrics and Gynecology Hospital of Fudan University	Researcher
Wenjing Diao	Obstetrics and Gynecology Hospital of Fudan University	Researcher
Ming Du	Obstetrics and Gynecology Hospital of Fudan University	Researcher
Hua Feng	Obstetrics and Gynecology Hospital of Fudan University	Researcher
Hongwei Zhang	Obstetrics and Gynecology Hospital of Fudan University	Researcher
Rong Jiang	Zhongshan Hospital Affiliated to Fudan University	Researcher
Jiarui Li	Xinhua Hospital Affiliated to Shanghai Jiao Tong University School of Medicine	Researcher
Xuhong Fang	Xinhua Hospital Affiliated to Shanghai Jiao Tong University School of Medicine	Researcher
Hailin Yu	Obstetrics and Gynecology Hospital of Fudan University	Researcher
Hao Feng	Obstetrics and Gynecology Hospital of Fudan University	Researcher
Ling Qiu	Obstetrics and Gynecology Hospital of Fudan University	Researcher
Shurong Zhu	Obstetrics and Gynecology Hospital of Fudan University	Researcher
Ming Li	Obstetrics and Gynecology Hospital of Fudan University	Researcher
Yanan You	Obstetrics and Gynecology Hospital of Fudan University	Researcher
Pengyao Jiang	Obstetrics and Gynecology Hospital of Fudan University	Researcher

6) The composition, roles and respective responsibilities of the trial sponsor, trial coordinating center, steering committee, endpoint determination committee, data management team and other teams.

The trial is sponsored by Shanghai Shenkang Hospital Development Center (contact: 021-96886), which is responsible for the management of hospital development in Shanghai and has oversight responsibility for this project. The study design, collection, management, analysis and interpretation of data, report writing, and publication of this trial are the responsibility of the main center of the Obstetrics and Gynecology Hospital of Fudan University, and the principle investigator has the final decision-making

OB&GY of Fudan University

authority. The ethics committee and the independent data monitoring body have the right of supervision.

This study is conducted by Hua Jiang and Xin Wu of the Obstetrics and Gynecology Hospital of Fudan University, who are responsible for the study design, implementation, and management. Weili Yan and Yan Du are responsible for statistical analysis, and quality control of the implementation. The all-round implementation rules have been developed and validated previously, and can be applied normally. The surgical team of this study consists of several groups in national or regional center hospitals, including: Hua Jiang and Xin Wu's team, Zhiling Zhu's team, Weiguo Hu's team and Xiaohong Xue'steam in the Obstetrics and Gynecology Hospital of Fudan University; Rongyu Zang, Libing Xiang, and Jiangrong Yu's team in Zhongshan Hospital of Fudan University; Wen Di and Weihua Lou's team in Renji Hospital of Shanghai Jiao Tong University; Xipeng Wang, Jiarui Li and Xuhong Fang's team in Xinhua Hospital of Shanghai Jiao Tong University; the team of Zhu Tao from Taizhou Cancer Hospital (Taizhou Hospital Affiliated Cancer Hospital of the University of Chinese Academy of Sciences); and the team of Yuyang Zhang from the First Affiliated Hospital of Wenzhou Medical University. In terms of statistical analysis, Weili Yan's team from CTU, Clinical Trial Center of the Children's Hospital of Fudan University is the randomized clinical research team (composed of epidemiologists and statisticians), responsible for statistical analysis, drawing graphs and explaining statistical results. Xin Wu, Tao Zhu, and Libing Xiang form the surgical quality control core team, are responsible for surgical quality control. Yan Du, as an epidemiologist, is responsible for the quality control of clinical study implementation.

The main center and sub-centers of this study each established an internal staff organization structure. The head of the main center regularly convenes the head of the sub-center for phase meetings. Each center has a fixed information contact person, who is managed by the main center. The information coordinator of the main center is responsible for collecting and releasing data, and receiving feedback from the sub-centers at any time, coordinating and handling temporary situations that arise in the work of the sub-centers, and ensuring that the progress of each center in the research is consistent.

The endpoint determination committee consists of the Co-PIs of each center, the ethics committee, the data inspection committee, and key members of the data management team to make appropriate determinations based on trial progress and analysis.

II. Background

2.1 Research Background

2.1.1 Overview of Cervical Cancer

Cervical cancer is one of the major malignancies that threaten women's health and lives worldwide, and it is currently the 4th most prevalent malignancy in women[1]. The

OB&GY of Fudan University

National Comprehensive Cancer Network (NCCN) guidelines for cervical cancer state that 528,000 new cases of cervical cancer occurred worldwide in 2012, and 85% of cervical cancers occur in developing countries. Persistent HPV infection is an important risk factor in the development of cervical cancer. Other high-risk factors for cervical cancer include early sexual intercourse, multiple sexual partners, multiple pregnancies, multiple births, smoking, long-term oral contraceptive use, and sexually transmitted diseases. The factors such as early sexual intercourse and multiple sexual partners are important synergistic factors for HPV infection. With the increase of HPV infection, the incidence of cervical cancer is increasing and the age becomes younger. HPV vaccination is the main strategy to prevent HPV-related cervical cancer. According to the WHO Global Cervical Cancer Elimination Program, HPV vaccination is one of the important components of the Program. However, due to relatively late implementation of HPV vaccine in China, the incidence and mortality of cervical cancer in China have shown increasing trends, with around 110,000 new cases and 50,000 deaths of cervical cancer in China in 2018, accounting for about 20% and 16% of the total global incidence and deaths, respectively. Among cervical cancer patients, the young age group (≤35 years old) accounts for 8.5%-12.6% of cervical cancer patients, and the survival benefits and quality of life of this group need particular attention.

Squamous cell carcinoma of the cervix accounts for approximately 80% of cervical cancers and adenocarcinoma for roughly 20%. In developed countries, the incidence and mortality of cervical squamous cell carcinoma have decreased significantly due to effective cervical cytologic screening. However, the incidence of cervical adenocarcinoma has gradually increased over the past 30 years probably due to insensitivity to cytologic screening, while the early identification of cervical adenocarcinoma may be improved if HPV testing is used as a screening method for cervical cancer; HPV vaccination may reduce not only the incidence of cervical squamous cell carcinoma but also the incidence of cervical adenocarcinoma[2, 3]. In addition to squamous and adenocarcinoma, cervical malignancies include other specific pathological types: small cell neuroendocrine carcinoma of the cervix, microbiased adenocarcinoma of the cervix, and malignant melanoma of the cervix.

Cervical cancer can infiltrate through and directly spread to the parametrial tissues, vagina, uterine corpus and adjacent organs (bladder and rectum) or through lymphatic vessels to regional lymph nodes such as closed foramen, external and internal iliac lymph nodes and then to the common iliac and para-abdominal aortic lymph nodes. The status of lymph nodes correlates with the prognosis of cervical cancer. Previously, FIGO staging of cervical cancer was clinical staging, and surgical pathological staging was not used. In 2018, FIGO updated the staging system of cervical cancer to include preoperative imaging and postoperative lymph node status. In addition, the American Joint Committee on Cancer (AJCC) also updated the TNM staging system of cervical cancer in 2018, so the FIGO 2018 and AJCC (2021) TNM staging system are both used in this study [4].

OB&GY of Fudan University

2.1.2 Treatment of cervical cancer

The main treatment methods of cervical cancer include surgery and radiotherapy. While surgery is the treatment of choice for early-stage cervical cancer (including stages IA1, IA2, IB1, IB2, and IIA1), it can also be used for the initial treatment of stages IB3 and IIA2. The main treatment modalities for cervical cancer are as follows:

- Radical cervical cancer surgery + pelvic lymph node dissection ± abdominal para-aortic lymph node dissection ± adjuvant therapy
- Radical radiotherapy
- Extensive hysterectomy (capable of IA2 to IB1 stage, preserving fertility)
- Cervical conization (stage IA1, preserving fertility)

The most appropriate treatment for cervical cancer requires consideration of a combination of patient age, fertility requirements, cervical cancer stage, comorbidities, patient and physician preferences, and the presence of risk factors associated with recurrence in the postoperative pathology report. NCCN recommends concurrent radiotherapy as the primary treatment for stage IB3 to IVA cervical cancer.

2.2.1 Surgical treatment

Radical cervical cancer surgery is the main procedure for the treatment of early-stage cervical cancer and is mainly applicable to cervical cancer above stage IA1 (including IA1 with LVSI, IA2, IB1, IB2, IIA1; and IB3 and IIA2). Compared with standard total hysterectomy, radical hysterectomy for cervical cancer has higher surgical difficulty and corresponding complications because of the larger surgical scope. Abdominal radical hysterectomy for cervical cancer has a history of more than 120 years, and the surgical approach has become very mature, while minimally invasive radical hysterectomy for cervical cancer has also become increasingly mature after nearly 20 years of development. Compared with traditional Abdominal Radical Hysterectomy (ARH), minimally invasive radical hysterectomy has unique advantages, such as less abdominal wall trauma, less pain, clearer vision and less bleeding, less interference with the intestine, and lower postoperative infection rate. Therefore, the 2014 NCCN guideline recommends laparoscopic radical hysterectomy (LRH) to perform radical cervical cancer surgery. Modern standard surgery includes laparoscopic surgery (divided into conventional laparoscopic surgery and robotic-assisted laparoscopic surgery, or multiport laparoscopic surgery and single-port laparoscopic surgery), open surgery, and transvaginal surgery. Because these three procedures are performed on different routes with the same extent of resection, it is intuitively assumed that the results of these three procedures are the same, and therefore most previous studies have focused on operative time, intraoperative bleeding, length of hospital stay, and cost, rather than survival indicators such as progression free survival (PFS) and overall Survival (OS). Only a few previous small

OBGY of Fudan University

studies have evaluated the survival benefit of Laparoscopic Radical Hysterectomy (LRH) for cervical cancer: in terms of PFS [5-10], LRH was not inferior to ARH, while laparoscopy was superior to open surgery in terms of operative time, intraoperative bleeding, and length of hospital stay. In November of 2018, the New England Journal of Medicine published two research reports comparing abdominal and minimally invasive radical cervical cancer surgery in the same period, which have shocked the entire gynecology field. The retrospective cohort study concluded [11] that over a median follow-up of 45 months, the 4-year mortality rate was 9.1% for women who underwent minimally invasive surgery and 5.3% for women who underwent open surgery (hazard ratio 1.65; 95% confidence interval 1.22-2.22); prior to the use of minimally invasive radical hysterectomy (i.e., from 2000 to 2006), the 4-year overall survival rate for women undergoing radical hysterectomy remained stable; while the overall survival rate decreased by 0.8% (95% CI, 0.3-1.4) per year after 2006 with the application of minimally invasive surgery,. Another study published in the New England Journal of Medicine in late 2018 (the Laparoscopic Approach to Cervical Cancer, LACC trial) was a Randomized Clinical Trial (RCT), which showed that the 4.5-year PFS rate was 86.0% in the minimally invasive surgery group and 96.5% in the open surgery group, with a difference of -10.6 percentage points (95% CI -16.4 to -4.7); minimally invasive surgery was associated with lower disease-free survival (PFS) compared with open surgery [(3-year PFS was 91.2% vs. 97.1%; hazard ratio for relapse or death of cervical cancer was 3.74; 95% CI (1.63 to 8.58)] [12]. Both studies suggest that minimally invasive radical cervical surgery is associated with lower PFS and OS rates compared with open surgery. Following the publication of these studies, the version 3.2019 of the NCCN guidelines recommended open surgery as the standard procedure for cervical cancer, while laparoscopic surgery is no longer recommended, which has caused a huge impact on the gynecology community.

Lymph node resection

Cervical cancer not only involves adjacent organs through direct infiltration, but also can metastasize to the pelvis and para-aortic lymph nodes through lymphatic vessels. Therefore, lymph node dissection is usually performed at the same time as radical cervical cancer surgery. The extent of lymph node dissection depends on the degree of disease progression.

For stage IA1 cervical cancer without LVSI, the risk of lymph node metastasis is very low, so lymph node dissection is not required. For stage IA1 cervical cancer with

OB&GY of Fudan University

LVSI, intraoperative evaluation of lymph node status and lymph node dissection or sentinel lymph node biopsy are required.

For stage IA2 cervical cancer, modified radical cervical cancer surgery + bilateral pelvic lymph node dissection is recommended. If preoperative imaging assessment of pelvic lymph node metastasis is suscepted, simultaneous abdominal para-aortic lymph node dissection is recommended.

For stage IB1, IB2, and IIA1 cervical cancer, the preferred surgical procedure is radical cervical cancer surgery + bilateral pelvic lymph node dissection ± abdominal para-aortic lymph node dissection. It is found that in IB-IIB cervical cancer, pelvic lymph node metastasis, tumor >2cm and common iliac lymph node metastasis are closely related to abdominal para-aortic lymph node metastasis. Therefore, for patients with suspected pelvic lymph node metastasis, parietal aortic lymph node dissection should be performed intraoperatively [13, 14].

For stage IB3 and IIA2 cervical cancer, the NCCN guidelines recommend simultaneous radiotherapy (Class I evidence) as the preferred treatment; it is controversial whether surgery is feasible for stage IB3 and IIA2 cervical cancer. However, some oncologists advocate surgery since stage IB3 and IIA2 tumors are larger (>4 cm) and have a higher risk of metastasis to the para-aortic lymph nodes. So if surgery is performed, radical cervical cancer surgery + bilateral pelvic lymph node dissection + abdominal para-aortic lymph node dissection should be performed.

Sentinel Lymph Node Mapping (SLN)

Recent studies have shown certain significance of SLN in early-stage cervical cancer. A meta-analysis has shown that the detection rate of sentinel lymph node mapping biopsy is 89-92% and the sensitivity is 89-90% [15, 16]. At the same time, studies have shown that sentinel lymph node mapping biopsy has some limitations, and intraoperative evaluation of sentinel lymph node methods (e.g., freezing or cell blotting) may miss micro-metastases in sentinel lymph nodes or isolated tumor cell metastases [17-19]. It has been suggested that the sensitivity of sentinel lymph node biopsy seems to be higher in cervical cancer with tumor size ≤ 2 cm [20, 21]; while the approach of sentinel lymph node hyperstaging improves the detection of lymph node micrometastases[22, 23].

Surgery to preserve fertility

Cervical conization for stage IA1 cervical cancer

For patients with stage IA1 cervical cancer with fertility preservation requirements, cervical conization ± pelvic lymph node dissection is recommended [24, 25]. The aim of cervical conization is to ensure negative margins [absence of invasive cancer and

OB&GY of Fudan University

high-grade squamous intraepithelial lesions (HSIL)]. For those with negative margins and no LVSI, postoperative observation and follow-up is sufficient; whereas in cases with positive margins, a second conization may be considered to clarify the depth of infiltration (to exclude stage IA2/IB) or to perform radical cervical hysterectomy. For stage IA1 with LVSI, cervical conization (negative margins) + laparoscopic sentinel lymph node mapping biopsy/pelvic lymph node dissection or radical cervical hysterectomy + SLN/pelvic lymph node dissection may be performed.

Radical cervical hysterectomy for stage IA2/IB1 cervical cancer

Patients with stage IA2 cervical cancer who have fertility preservation requirements are recommended to undergo radical hysterectomy + pelvic lymph node dissection, or can consider radical hysterectomy + SLN. If cervical cone margins are negative, pelvic lymph node dissection can also be performed to assess lymph node status. If lymph node status is negative, follow-up can be performed. Patients with stage IB1 cervical cancer who have fertility preservation requirements are recommended to undergo radical hysterectomy + pelvic lymph node dissection ±para-aortic lymph node dissection [26-28].

2.2.2 Indications for adjuvant therapy after cervical cancer surgery

The need for adjuvant therapy after radical surgery for cervical cancer is determined by the stage of the disease and surgical pathological factors. Patients with stage IA2, IB1 or IIA1 who have no postoperative lymph node metastases, negative surgical margins, negative parametrium and no other risk factors for relapse (Sedlis criteria) do not need adjuvant therapy after surgery and should be followed0up regularly. However, if any replase risk factors are present, postoperative adjuvant therapy is recommended.

High risk disease cervical cancer

After radical cervical surgery, patients with three high-risk factors (positive lymph nodes, positive surgical margins, and positive parametrium) are recommended to receive postoperative adjuvant therapy: external-beam radiation therapy (EBRT) + synchronous platinum-based chemotherapy + vaginal brachytherapy. Vaginal brachytherapy is a very effective local thrust for patients with positive vaginal margins; while synchronized radiotherapy significantly improves OS in patients with all three high-risk factors after radical cervical surgery for early-stage cervical cancer [29].

Intermediate-risk disease cervical cancer

Postoperative adjuvant pelvic EBRT+-synchronous platinum-based chemotherapy is required after radical cervical surgery for cervical cancer in the absence of high-risk

OB&GY of Fudan University

factors, but in the presence of large tumor size, deep interstitial infiltration, and LVSI (see NCCN guidelines, Sedlis criteria) [30-32].

2.2.3 Radical radiotherapy

Radical radiotherapy is indicated for the treatment of stage IB3, stage IIA2, and advanced (stage IIB-IVA) cervical cancer.

For stage IB3 or IIA2 cervical cancer, the NCCN recommendation (Class 1 evidence) is that concurrent radiotherapy is preferred: radical EBRT + concurrent platinum-based chemotherapy + vaginal brachytherapy. For those with stage IB1, IB2, or IIA cervical cancer that cannot tolerate surgery, pelvic EBRT + vaginal brachytherapy ± platinum-based concurrent chemotherapy can be performed.

For patients with advanced cervical cancer receiving radical radiotherapy, the target area of radiotherapy is critical, which is determined by pelvic and abdominal para-aortic lymph node metastases. Magnetic resonance imaging (MRI) helps to describe the extent of localized lesions and aids in radiotherapy planning. However, fine needle aspiration biopsy may be considered for suspicious imaging presentations outside the uterus to clarify the diagnosis. Surgical evaluation of lymph node status (i.e., extraperitoneal or laparoscopic lymph node dissection) is also an option for these patients (category 2B), and surgical evaluation can also detect microscopic lymph node metastases that are not detectable on imaging [33, 34]. For those without lymph node metastases or lesions confined to the pelvis, pelvic EBRT + synchronous platinum-based chemotherapy + vaginal brachytherapy is feasible (class 1 evidence) [35] [36]. For patients with positive pelvic and para-aortic lymph nodes, imaging is recommended to assess for distant metastases. If there are no distant metastases, pelvic extension field EBRT + simultaneous platinum-containing chemotherapy + vaginal brachytherapy is recommended; in the presence of distant metastases and positive para-aortic lymph nodes, systemic chemotherapy ±individualized EBRT is recommended [37].

Either pelvic radiotherapy or radiotherapy could lead to ovarian failure in premenopausal women. To protect endocrine function of the ovaries, young women (under 45 years of age, squamous carcinoma) can undergo ovarian transposition surgery before pelvic radiotherapy [38-40].

2.2.4 Surgery vs. radiotherapy

Both radical cervical surgery and radical radiotherapy are main treatments for early-stage cervical cancer; while radical radiotherapy/radiochemotherapy is recommended for advanced-stage cervical cancer. Therefore, the choice of treatment

OB&GY of Fudan University

modality needs to take into account disease stage, fertility requirements, comorbidities, quality of life, and the personal preference of both physicians and patients. Surgery is indicated for patients with early stage cervical cancer, especially for those with fertility preservation requirements. For patients with stage IA, IB1, IB2, and stage IIA1 cervical cancer, the NCCN guidelines recommended surgery for the following reasons:

. Young women can preserve ovarian function.

. The possibility of preserving vaginal function after surgery is higher compared to radiotherapy.

. Intraoperative lymph node dissection can be performed simultaneously during the surgical treatment, if postoperative radiotherapy is needed. Individualized radiotherapy formulation can be achieved.

For stage IB3 and IIA2 cervical cancer, the NCCN guidelines recommend concurrent chemoradiotherapy (CCRT): radical EBRT + concurrent platinum-based chemotherapy + vaginal brachytherapy. After synchronous radiochemotherapy, patients may experience vaginal function loss, ovarian function decline, and poor quality of life. A proportion of these patients are young women. Currently in China, there are issues of uneven distribution of radiotherapy resources and imbalance in the quality of radiotherapy. Therefore, patients with IB3 and IIA2 stage are also included as the target population of receiving radical treatment in this study.

2.3 Prognosis

The main prognostic factors affecting squamous cervical cancer include tumor stage, lymph node status, tumor size, depth of cervical interstitial infiltration, LVSI (lymphovascular interstitial infiltration), and histological type and grading of the tumor. Tumor stage is the most important prognostic factor, followed by lymph node status. After radical cervical cancer surgery + lymph node dissection for clinical stage IB or IIA patients, the 5-year OS was 88-96% for those without lymph node metastasis, while it was 64-74% for those with lymph node metastasis [41, 42].The 2018 LACC trial found that in stage IA1 with LVSI, IA2 and IB1 cervical cancer patients, the PFS and OS rates of patients receiving minimally invasive radical cervical cancer surgery were lower than those of receiving open surgery, with a PFS rate of 91.2% vs. 97.1% at 3 years, and an OS rate of 93.8% vs. 99.0% at 3 years for minimally invasive surgery versus open surgery, respectively [12]. In contrast, another retrospective study in 2018 found [11] that the relative survival of patients with cervical cancer decreased by 0.8% per year in the 4 years since the use of minimally invasive surgery began in 2006. Therefore, the NCCN guidelines no longer recommend minimally invasive radical cervical cancer surgery. To

OB&GY of Fudan University

sum up, the 5-year overall survival rate for stage I cervical cancer is about 90% and for stage II cervical cancer is about 60%.

2.4 Laparoscopic surgery

Laparoscopic radical cervical cancer surgery was originally reported by Canis [43] and Nezhat[44] et al. In the last two decades, laparoscopic surgery has become increasingly sophisticated. In addition to traditional laparoscopic surgery, robotic-assisted surgery has been derived. Most studies have shown that laparoscopic surgery has less intraoperative bleeding and shorter hospital stay, and some studies have reported that the PFS rate of LRH was not inferior to that of ARH [5-10]. Therefore, laparoscopic surgery has become the main treatment for early-stage cervical cancer in many countries, and the 2014 NCCN guidelines recommended a laparoscopic approach for radical cervical cancer. In most of the prestige hospitals in China, laparoscopic radical cervical cancer treatment accounts for more than 90% of the cases.

As previously mentioned, the LACC trial and the retrospective study published in the New England Journal of Medicine in late 2018 caused a huge impact in the medical community when they concluded that both PFS and OS rates for patients undergoing minimally invasive radical cervical cancer treatment were lower than those undergoing abdominal radical cervical cancer treatment. Following the publication of these articles, the version 3.2019 NCCN guidelines have recommended open surgery as the standard procedure for cervical cancer, and laparoscopic surgery is no longer recommended.

However, at our hospital (Obstetrics and Gynecology Hospital of Fudan University), about 1,500-2,000 cervical cancer surgeries were performed each year over the past 8 years. According to our data, for laparoscopic radical cervical cancer surgery, the 5-year OS rates are 100% in stage IA2 and 96.93% in stage IB1 patients, which are similar to open surgery in the LACC trial. In addition, data from endometrial cancer have shown that laparoscopy is superior to open surgery in terms of both recent surgery-related complications and survival benefits, so NCCN guidelines recommend laparoscopic surgery as the standard procedure for endometrial cancer. Until now, there is only one RCT (the LACC trial) that compared minimally invasive and open radical cervical cancer treatments. In view of the results of the LACC trial, should we completely abandon minimally invasive surgery for cervical cancer, or are we looking for the root cause and improvement methods to promote medical development? Therefore, we propose to design this study in accordance with the high requirements of our ability and intraoperative tumor-free principle, with the aim of further comparing LRH (or robotic-assisted) and ARH in patients with early-stage cervical cancer: IA1 with intravascular infiltration

OB&GY of Fudan University

(LVSI), stage IA2; IB1, IB2, IIA1; stage IB3, IIA2 in terms of efficacy as well as quality of life.

2.5 Quality of life

Quality of life studies allow evaluate and comparison of surgical outcomes from the patient's perspective and are therefore an extremely important assessment endpoint. The qualityof life survey used in this phase III clinical trial will include the use of the EORTC Quality of Life Measurement Scale QLQC30 (V3.0), the EORTC QLQ-CX24 scale, and the PRO 147 (V 1.0), a patient-reported clinical outcome evaluation scale for cervical cancer developed in-house by the research team.

References:

[1]. Bray, F., et al., Global cancer statistics 2018: GLOBOCAN estimates of incidence and mortality worldwide for 36 cancers in 185 countries. CA Cancer J Clin, 2018. 68(6): p. 394-424.

[2]. Castellsague, X., et al., Worldwide human papillomavirus etiology of cervical adenocarcinoma and its cofactors: implications for screening and prevention. J Natl Cancer Inst, 2006. 98(5): p. 303-15.

[3]. Dahlstrom, L.A., et al., Prospective study of human papillomavirus and risk of cervical adenocarcinoma. Int J Cancer, 2010. 127(8): p. 1923-30.

[4]. Olawaiye, A.B., et al., The new (Version 9) American Joint Committee on Cancer tumor, node, metastasis staging for cervical cancer. CA Cancer J Clin, 2021.

[5]. Aarts, J.W., et al., Surgical approach to hysterectomy for benign gynaecological disease. Cochrane Database Syst Rev, 2015(8): p. CD003677.

[6]. Magrina, J.F., et al., Robotic radical hysterectomy: comparison with laparoscopy and laparotomy. Gynecol Oncol, 2008. 109(1): p. 86-91.

[7]. Geisler, J.P., et al., Robotically assisted laparoscopic radical hysterectomy compared with open radical hysterectomy. Int J Gynecol Cancer, 2010. 20(3): p. 438-42.

[8]. Roque, D.R., W.Z. Wysham and J.T. Soper, The surgical management of cervical cancer: an overview and literature review. ObstetGynecolSurv, 2014. 69(7): p. 426-41.

[9]. Nam, J.H., et al., Laparoscopic versus open radical hysterectomy in early-stage cervical cancer: long-term survival outcomes in a matched cohort study. Ann Oncol, 2012. 23(4): p. 903-11.

[10]. Bogani, G., et al., Laparoscopic versus open abdominal management of cervical cancer: long-term results from a propensity-matched analysis. J Minim Invasive Gynecol, 2014. 21(5): p. 857-62.

[11]. Melamed, A., et al., Survival after Minimally Invasive Radical Hysterectomy for Early-Stage Cervical Cancer. N Engl J Med, 2018. 379(20): p. 1905-1914.

[12]. Ramirez, P.T., et al., Minimally Invasive versus Abdominal Radical Hysterectomy for Cervical Cancer. N Engl J Med, 2018. 379(20): p. 1895-1904.

OB&GY of Fudan University

[13]. Sakuragi, N., et al., Incidence and distribution pattern of pelvic and paraaortic lymph node metastasis in patients with Stages IB, IIA, and IIB cervical carcinoma treated with radical hysterectomy. Cancer, 1999. 85(7): p. 1547-54.

[14]. Huang, H., et al., Metastasis to deep obturator and para-aortic lymph nodes in 649 patients with cervical carcinoma. Eur J Surg Oncol, 2011. 37(11): p. 978-83.

[15]. Wu, Y., et al., Sentinel lymph node biopsy in cervical cancer: A meta-analysis. Mol Clin Oncol, 2013. 1(6): p. 1025-1030.

[16]. Kadkhodayan, S., et al., Sentinel node biopsy for lymph nodal staging of uterine cervix cancer: a systematic review and meta-analysis of the pertinent literature. Eur J Surg Oncol, 2015. 41(1): p. 1-20.

[17]. Bats, A.S., et al., Diagnostic value of intraoperative examination of sentinel lymph node in early cervical cancer: a prospective, multicenter study. Gynecol Oncol, 2011. 123(2): p. 230-5.

[18]. Fader, A.N., et al., Sentinel lymph node biopsy in early-stage cervical cancer: utility of intraoperative versus postoperative assessment. Gynecol Oncol, 2008. 111(1): p. 13-7.

[19]. Slama, J., et al., High false negative rate of frozen section examination of sentinel lymph nodes in patients with cervical cancer. Gynecol Oncol, 2013. 129(2): p. 384-8.

[20]. Cormier, B., et al., Establishing a sentinel lymph node mapping algorithm for the treatment of early cervical cancer. Gynecol Oncol, 2011. 122(2): p. 275-80.

[21]. Eiriksson, L.R. and A. Covens, Sentinel lymph node mapping in cervical cancer: the future? BJOG, 2012. 119(2): p. 129-33.

[22]. Cibula, D., et al., Bilateral ultrastaging of sentinel lymph node in cervical cancer: Lowering the false-negative rate and improving the detection of micrometastasis. Gynecol Oncol, 2012. 127(3): p. 462-6.

[23]. Galic, V., et al., Prognostic significance of adenocarcinoma histology in women with cervical cancer. Gynecol Oncol, 2012. 125(2): p. 287-91.

[24]. Andikyan, V., et al., Cervical conization and sentinel lymph node mapping in the treatment of stage I cervical cancer: is less enough? Int J Gynecol Cancer, 2014. 24(1): p. 113-7.

[25]. Wright, J.D., et al., Fertility-conserving surgery for young women with stage IA1 cervical cancer: safety and access. ObstetGynecol, 2010. 115(3): p. 585-590.

[26]. Bernardini, M., et al., Pregnancy outcomes in patients after radical trachelectomy. Am J ObstetGynecol, 2003. 189(5): p. 1378-82.

[27]. Diaz, J.P., et al., Oncologic outcome of fertility-sparing radical trachelectomy versus radical hysterectomy for stage IB1 cervical carcinoma. Gynecol Oncol, 2008. 111(2): p. 255-60.

[28]. Raju, S.K., et al., Fertility-sparing surgery for early cervical cancer-approach to less radical surgery. Int J Gynecol Cancer, 2012. 22(2): p. 311-7.

[29]. Peters, W.R., et al., Concurrent chemotherapy and pelvic radiation therapy compared with pelvic radiation therapy alone as adjuvant therapy after radical surgery in high-risk early-stage cancer of the cervix. J Clin Oncol, 2000. 18(8): p. 1606-13.

[30]. Sedlis, A., et al., A randomized trial of pelvic radiation therapy versus no further therapy in selected patients with stage IB carcinoma of the cervix after radical hysterectomy and pelvic lymphadenectomy: A Gynecologic Oncology Group Study. Gynecol Oncol, 1999. 73(2): p.

OB&GY of Fudan University

177-83.
[31]. Rotman, M., et al., A phase III randomized trial of postoperative pelvic irradiation in Stage IB cervical carcinoma with poor prognostic features: follow-up of a gynecologic oncology group study. Int J Radiat Oncol Biol Phys, 2006. 65(1): p. 169-76.
[32]. Chernofsky, M.R., et al., Influence of quantity of lymph vascular space invasion on time to recurrence in women with early-stage squamous cancer of the cervix. Gynecol Oncol, 2006. 100(2): p. 288-93.
[33]. Kohler, C., et al., Perioperative morbidity and rate of upstaging after laparoscopic staging for patients with locally advanced cervical cancer: results of a prospective randomized trial. Am J ObstetGynecol, 2015. 213(4): p. 503.e1-7.
[34]. Goff, B.A., et al., Impact of surgical staging in women with locally advanced cervical cancer. Gynecol Oncol, 1999. 74(3): p. 436-42.
[35]. Rose, P.G., Combination therapy: New treatment paradigm for locally advanced cervical cancer? Nat Rev Clin Oncol, 2011. 8(7): p. 388-90.
[36]. Whitney, C.W., et al., Randomized comparison of fluorouracil plus cisplatin versus hydroxyurea as an adjunct to radiation therapy in stage IIB-IVA carcinoma of the cervix with negative para-aortic lymph nodes: a Gynecologic Oncology Group and Southwest Oncology Group study. J Clin Oncol, 1999. 17(5): p. 1339-48.
[37]. Lutz, S.T., et al., A review of hypofractionated palliative radiotherapy. Cancer, 2007. 109(8): p. 1462-70.
[38]. Wo, J.Y. and A.N. Viswanathan, Impact of radiotherapy on fertility, pregnancy, and neonatal outcomes in female cancer patients. Int J Radiat Oncol Biol Phys, 2009. 73(5): p. 1304-12.
[39]. Pahisa, J., et al., Laparoscopic ovarian transposition in patients with early cervical cancer. Int J Gynecol Cancer, 2008. 18(3): p. 584-9.
[40]. Morice, P., et al., Ovarian transposition for patients with cervical carcinoma treated by radiosurgical combination. FertilSteril, 2000. 74(4): p. 743-8.
[41]. Delgado, G., et al., Prospective surgical-pathological study of disease-free interval in patients with stage IB squamous cell carcinoma of the cervix: a Gynecologic Oncology Group study. Gynecol Oncol, 1990. 38(3): p. 352-7.
[42]. Averette, H.E., et al., Radical hysterectomy for invasive cervical cancer. A 25-year prospective experience with the Miami technique. Cancer, 1993. 71(4 Suppl): p. 1422-37.
[43]. Canis, M., et al., [Does endoscopic surgery have a role in radical surgery of cancer of the cervix uteri?]. J GynecolObstet Biol Reprod (Paris), 1990. 19(7): p. 921.
[44]. Nezhat, C.R., et al., Laparoscopic radical hysterectomy with paraaortic and pelvic node dissection. Am J ObstetGynecol, 1992. 166(3): p. 864-5.

OB&GY of Fudan University

2.2Research Objectives

2.2.1 Main study objectives

To compare the 5-year overall survival (OS) of patients undergoing Laparoscopic Radical Hysterectomy (LRH) versus Abdominal Radical Hysterectomy (ARH) for

cervical cancer.

2.2.2 Secondary research objectives

To compare the 5-year progression free survival (PFS) of the two groups of patients with different surgical procedures.

To compare the relapse rate and relapse pattern between the two groups of patients with different surgical procedures.

To compare the functional status of each organ in the two groups.

To compare the effects of the two different surgical procedures on quality of life of the patients in the two groups.

To compare the difference in cost effectiveness between the two groups of patients.

To compare the amount of surgical bleeding, the incidence of surgery-related diseases, and intraoperative and postoperative complications between the two groups.

2.3 Research Design

STAGE1

The publication of the LACC trial has caused a huge impact in the gynecology field, and the version 3.2019 NCCN guidelines no longer recommended laparoscopic surgery for patients with cervical cancer. However, the investigators reviewed data from our hospital for the last 8 years of laparoscopic radical cervical cancer surgery, which showed different results from the LACC trial. During the Global Clinical Scholar Research Training (GCSRT) program hosted by Harvard Medical School, the leader of the main center discussed with local clinical research experts about the ethical feasibility of redesigning the RCT study to validate the topic, and in the first draft of this project design was completed as part of the Capstone Project course plan, and the study design was recommended in the end-of-semester presentation.

STAGE2

According to the trial design, after the study passed by ethics, the research team started to try the protocol according to the design process and successfully recruited 84 subjects. At the beginning of the study design, the study mixed cervical cancer stages IA1 with LVSI, IA2, IB1, IB2, IIA1, IB3, and IIA2 in one trial. However, as the trial progressed, we found that the span of the trial stages was too large for a specific assessment of each stage and for guiding the specific procedure for each stage. Also, when we submitted the protocol, we received the same suggestion from the review responses.

STAGE3

This project is divided into three parallel studies based on the staging, LAUNCH 1 (LA1 with LVSI, IA2), LAUNCH 2 (IB1, IB2, IIA1), and LAUNCH 3 (IB3, IIA2). Previously we conducted a single-center study (study protocol registration number: ChiCTR1900025946) which included all above mentioned stages. There are 84 patients

of Fudan University

enrolled in that study which will not be included in the current project, since those patients do not meet the randomization strategy of the current project.

STAGE4

This study intends to sample and collect intraoperative tumor tissues from tumor patients, and to conduct basic science research on the evaluation of genetic testing for specific tumor stages, which can be used to guide later treatments such as targeted or immune therapies. At the same time, we will conduct relevant histological studies on the molecular tissues related to tumors in order to find the pathogenesis pattern that may guide treatment.

III. Research methods

1. Study setup

This project is a multicenter study led by the Obstetrics and Gynecology Hospital of Fudan University, which is one of the first national clinical class I key specialties in China and a key discipline of the Ministry of Education. The Obstetrics and Gynecology Hospital of Fudan University undertakes the task of diagnosis and treatment of malignant tumors of the female reproductive system nationwide, with a leading annual volume of cervical cancer surgery and treatment in China. The clinical centers of this study includes Obstetrics and Gynecology Hospital of Fudan University (main center), Zhongshan Hospitalaffiliated to Fudan University, Renji Hospital of Shanghai JiaoTong University, Xinhua Hospital of Shanghai Jiao Tong University, Taizhou Hospital Affiliated Cancer Hospital of the University of Chinese Academy of Sciences and the First Hospital of Wenzhou Medical University (sub-centers). The CTU of the Clinical Trial Center of the Children's Hospital of Fudan University is responsible for statistical analyses. All these have provided adequate technical support and guarantee for the study.

2. Qualification criteria

2.1 Study subjects and study time

A total of 1104 patients diagnosed with cervical cancer (stage IB3 and IIA2) and meeting the enrollment criteria will be recruited from the aforementioned main center and sub-centers. This project is expected to be completed in 3 years (April 2021 to April 2024) from the date of formal ethical approval and completion of enrollment, and the patients will be followed-up for 5 years (April 2021 to April 2026).

2.2 Patient inclusion and exclusion criteria

Patient inclusion criteria:

a) Clinical diagnosis of squamous carcinoma of the cervix, adenocarcinoma, squamous adenocarcinoma (stage IB3 and IIA2).

b) Age $\geq$ 21 years and $\leq$ 70 years.

c) Surgery type C (refer to Q-M surgical staging)

OB&GY of Fudan University

d) Normal range of liver and kidney function, and blood count:
Hemoglobin > 60g/L;
Platelets > $70*10^9$/L;
Leukocytes > $3*10^9$/L;
Creatinine < 50mg/dL;
Transaminase abnormal indicators ≤ 3;
Maximum value of transaminases not exceeding 3 times the corresponding normal value.
e) No history of other malignancies.
f) Non-pregnancy.
g) Physical strength classification: Karnofsky score ≥ 60.
h) Voluntarily join the study, sign the informed consent form, are compliant and cooperative with the follow-up.
i) No psychiatric disorders and other serious immune system disorders (e.g. lupus erythematosus, myasthenia gravis, HIV infection, etc.)
(Note: Maximum diameter measurement of cervical lesion is based on PET-CT, CT, or MRI)

Patient exclusion criteria:

a) Contraindicated for various surgeries and cannot undergo surgery.
b) Have received pelvic/abdominal radiotherapy irradiation or neoadjuvant chemotherapy for cervical cancer.
c) With recurrent cervical cancer
d) With CT, MRI or PET-CT suggesting suspicious metastasis of pelvic lymph nodes with maximum diameter >2cm after further improvement of preoperative examination.

2.3 Requirements of investigators and surgeons

Surgeons involved in this project were selected from each participating hospital and are skilled in cervical cancer surgery. The lead surgeon must have experience in at least 50 surgeries each for both LRH and ARH and be able to provide the medical history of these cases. The principal investigator and members of the surgical quality control team will be on site to observe each surgeon's procedure and review the unedited video of the procedure (1 ARH and 1 LRH) provided by each surgeon for surgical quality measurement and documentation to ensure the extent of surgical resection and tumor-free management of each surgeon.

3. Interventions

3.1 Overall intervention plans

Minimally invasive radical cervical cancer treatment includes laparoscopic radical cervical cancer treatment (LRH) and robotic-assisted laparoscopic radical cervical cancer treatment, either of which can be chosen by the operator. This project focuses on the survival benefit of patients after minimally invasive and open radical cervical cancer

OB&GY of Fudan University

treatment, so radical cervical cancer treatment with preservation of reproductive function will not be included. For both open and minimally invasive radical cervical cancer surgery, the operation begins with a thorough abdominal exploration, including careful exploration of the diaphragm, and any metastatic lesions and metastatic sites should be described in detail in the operative record, with biopsy for confirmative diagnosis. If intra-abdominal lesions are found, radical cervical cancer surgery should not be used and should change to palliative treatment.

Pelvic lymph node dissection should be performed during radical cervical cancer surgery. Sentinel Lymph Node Mapping (SLN) is not included in this project, since there is no sufficient evidence for its sensitivity and specificity, and also the difficulty in achieving lymph node hyperstaging considering the large sample size of this project. If the tumor is ≥2 cm, or if the common iliac lymph node is positive for intraoperative freezing (optional), or if the preoperative evaluation of the paraaortic lymph nodes is positive, paraaortic lymph node dissection is preferred and followed by paraaortic lymph node biopsy. When clear the para-aortic lymph nodes, the upper border reaches the level of the inferior mesenteric artery is enough. The common iliac lymph nodes should be sent separately for examination, and the resection should include both sides of the common iliac vessels, with the upper border reaching the midpoint of the bifurcation of the abdominal aorta and the common iliac vessels and the lower border reaching the level of the bifurcation of the common iliac vessels.

If definite pelvic lymph node metastasis is found intraoperatively, continuation of pelvic lymph node dissection, and even radical cervical cancer surgery is not required, but abdominal para-aortic lymph node sampling is recommended to assess the degree of disease progression and to develop subsequent radiotherapy regimens. If surgery is continued, radical cervical cancer surgery + pelvic lymph node dissection + abdominal para-aortic lymph node dissection/biopsy is recommended.

For patients randomly assigned to LRH, the surgeon should perform laparoscopic/robotic-assisted radical laparoscopic cervical cancer surgery or perform laparoscopic-assisted radical transvaginal cervical cancer surgery.

In performing radical cervical cancer surgery, the surgeon should provide detailed descriptions of the special surgical instruments used in the laparoscopic/robotic-assisted laparoscopic group. In addition, the surgeon should document the following information in the operative record:

. Time of surgery: time from the start of radical cervical cancer surgery to the closure of all abdominal puncture sites or abdominal incisions.

. Amount of intraoperative blood loss.

. Intraoperative complications.

. If patients are randomized to the laparoscopic surgery group and are converted to open surgery intraoperatively, the reasons should be recorded.

OB&GY of Fudan University

. In case of robotic-assisted laparoscopic radical cervical cancer surgery, the time required to assemble and dock the robot should be recorded in the operative record, which should be deducted from the total operative time.

. If intraoperative parietal aortic lymph node dissection is performed simultaneously, the time required for this operation should be recorded in the operative record.

The surgical approach for stage IA1 with LVSI and stage IA2 is type B (modified radical hysterectomy with bilateral pelvic lymph node dissection, i.e., resection of 1-2 cm of the parametrium and 1-2 cm of the vagina) with para-aortic lymph node dissection if necessary (surgical staging according to FIGO 2018, surgical staging according to Querleu-Morrow staging).

For stage IB1, IB2, and IIA1, the surgical approach is typeC1 (radical cervical surgery with preservation of autonomic nerves + pelvic lymph node dissection, recommended for tumor diameter <2 cm) or type C2 (radical cervical surgery with bilateral pelvic lymph node dissection, resection of 3-4 cm of the parametrium and the upper 1/4 to 1/3 of the vagina) with abdominal para-aortic lymph node dissection if necessary (surgical staging according to FIGO 2018, staging according to Querleu-Morrow staging). The depth of parametrial tissue removed should be below the deep uterine vein, and if preoperative involvement of the vaginal wall is considered, 1-2 cm of parametrial tissue should be removed.

Stage IB3, IIA2 The surgical approach is type C2 (radical cervical cancer + bilateral pelvic lymph node dissection + para-aortic lymph node dissection, 3-4 cm of parametrium and upper 1/4 to 1/3 of the vagina) (surgical staging according to FIGO 2018, surgical staging according to Querleu-Morrow staging). Parametrial tissue should be removed to a depth below the deep uterine vein, and 1-2 cm of parametrial tissue should be removed if preoperative involvement of the vaginal wall is considered.

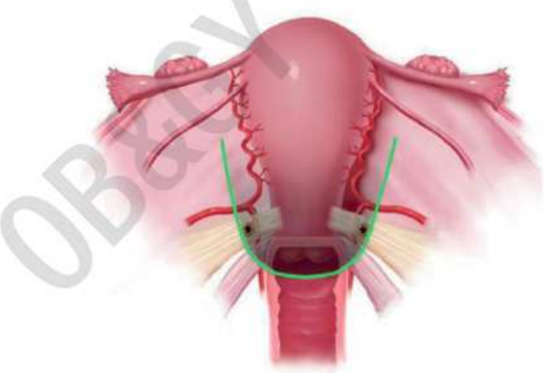

Figure 1A

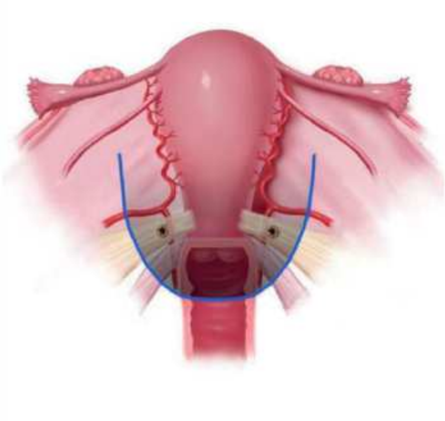

Figure 1B

OB&GY of Fudan University

Figure 1A. Type B: Surgical resection range of modified radical hysterectomy
Figure 1B. Type C2: Surgical resection range radical cervical cancer

The tumor-free principle during surgery includes:

1) Severing the vagina before vaginal closure by: closure of the vagina with an obturator; ligature ring ligation of the vagina; transvaginal suture of the vagina.

2) After laparoscopic ligation of the vagina, the vagina is irrigated with sterile water, and then the vagina is severed and sutured; after open surgery, the vagina is closed with renal clamps and the anterior vaginal wall is incised, gauze is inserted, then the posterior wall is severed and the vaginal stump is sterilized.

3) Encourage sharp and gentle excision, and try to avoid vigorous tearing and plucking movements.

4) Whole lymph nodes are removed without touching other parts and placed in a specimen bag for final removal; extensive hysterectomy is performed in whole and excessive straining is avoided intraoperatively.

5) Rinse the instruments that have contacted the tumor in the vagina and separate them from other instruments; if suspicious metastatic tissue is encountered in the pelvic and abdominal cavity, remove it directly into the specimen bag without contacting other parts after excision.

6) Aspirate the pelvic and abdominal cavity after full postoperative rinsing with sterile water, and avoid backflow of pelvic rinsing fluid into the upper abdomen when the head is low and the feet are high.

7) Do not lift the uterus. Use other methods such as silk thread to pull the uterine body.

3.2 Standard treatment plan:

Open radical hysterectomy for cervical cancer + pelvic lymph node dissection/para-aortic lymph node dissection

a) The preoperative prophylactic antibiotics are given at least 15 minutes before incision of the skin.
b) The sequential compression device (antithrombotic pressure pump) is installed.
c) The patient is placed in the supine position or bladder lithotomy position.
d) Considering the wide range of surgical instruments used in this type of surgery, the use of laparoscopic instruments depends on the operator's personal choice.
e) The incision is chosen as a median abdominal incision or a low transverse incision.

OB&GY of Fudan University

f) After entering the abdomen, a thorough abdominal exploration is performed, including careful exploration of the diaphragm. Any metastatic lesions and metastatic sites should be described in detail in the surgical record, and biopsy is performed to confirm the diagnosis.
g) The lateral pelvic peritoneum is opened and any enlarged, suspicious lymph nodes should be removed (if feasible) followed by intraoperative freezing. If these lymph nodes are positive, abdominal para-aortic lymph node biopsy should be performed, but radical cervical cancer surgery may be forgone and the treatment regimen for this patient will be switched to radical radiotherapy; or radical cervical cancer surgery + pelvic lymph node dissection + abdominal para-aortic lymph node dissection/biopsy may continue.
h) The radical cervical cancer surgery with or without bilateral salpingo-oophorectomy will be performed if there are no suscepted pelvic lymph nodes.
i) The Removal of ovaries: the ovaries may be preserved or removed, or ovarian transposition may be performed, depending on the subject's condition.
j) The scope of pelvic lymph node dissection: lymph nodes adjacent to the common iliac artery, external iliac artery, internal iliac artery and lymph nodes in the closed fossa should be removed.
k) The abdominal para-aortic lymph node dissection should reach the level of the inferior mesenteric artery.
l) Tumor-free principle: use open surgical kidney pedicle forceps to clamp the vagina close, then incision of the anterior vaginal wall, insertion of gauze, followed by severance of the posterior wall and disinfection of the vaginal stump.
m) The abdominal drainage tube is non-compulsory and can be chosen by each center.
n) The closure of the abdomen layers: closure of the fascia and closure of the skin.
o) Indwelling urine catheter.

3.3 Intervention treatment:

Laparoscopic or robot-assisted laparoscopic radical hysterectomy for cervical cancer + pelvic lymph node dissection/para-aortic lymph node dissection.

1) The preoperative prophylactic antibiotics are given at least 15 minutes before incision of the skin.
2) The sequential compression device (antithrombotic pressure pump) is installed.
3) The patient is placed in bladder lithotomy position with the upper arms flat on the sides of the body or on the chest.
4) The choice of laparoscopic instruments depends on the surgeon's personal preference,

OB&GY of Fudan University

considering the wide range of surgical instruments used for this type of procedure.

5) The approach to the abdomen and the number of puncture points for laparoscopic surgery depend on the operator's personal choice.
6) After entering the abdomen, a thorough abdominal exploration, including careful exploration of the diaphragm, should be performed first. Any metastatic lesions and metastatic sites should be described in detail in the operative record, and a biopsy should be performed to clarify the diagnosis.
7) The round ligament is separated in order to open the retroperitoneum. The lateral pelvic peritoneum is opened and any enlarged, suscepted lymph nodes should be resected (if feasible) followed by intraoperative cryopexy. If these lymph nodes are positive, abdominal para-aortic lymph node biopsy should be performed, but radical cervical cancer surgery may be forgone and the treatment plan will be changed to radical radiotherapy in this subject; or radical cervical cancer surgery + pelvic lymph node resection + abdominal para-aortic lymph node dissection/biopsy may be continued.
8) The radical cervical cancer surgery with or without bilateral salpingo-oophorectomy will be performed if there are no suscepted pelvic lymph nodes.
9) The removal of ovaries: the ovaries may be preserved or removed or ovarian transposition may be performed depending on the patient's specific situation.
10) The scope of pelvic lymph node resection: the lymph nodes adjacent to the common iliac artery, external iliac artery, internal iliac artery and the lymph nodes in the closed fossa should be removed.
11) The uterus is not lifted, and methods such as silk traction of the uterine body are used.
12) The uterine artery is identified and dissociatedat the point where the internal iliac artery divides from the uterine artery.
13) The parametrial tissue and the uterosacral ligament are excised to ensure adequate surgical margins. Paying attention to the tumor- free principle, the vagina is closed before severing it, which can be done by: closure of the vagina with an obturator; closure of the vagina with a ligature ring; and closure of the vagina with transvaginal sutures. After laparoscopic closure of the vagina, the vagina is irrigated with sterile water, after which the vagina is severed and the vaginal stump is sutured.
14) Systematic lymphadenectomy: removal of lymph nodes adjacent to the common iliac artery, external iliac artery, internal iliac artery and lymph nodes in the closed fossa ± parietal aortic lymph nodes (up to the level of the inferior mesenteric artery).
15) The intraoperative lymph nodes are removed by en bloc resection, without touching other parts, and will be placed in a specimen bag for final removal.

OB&GY of Fudan University

16) The postoperative pelvic and abdominal cavity will be suctioned after full flushing with sterile water, and the pelvic flushing fluid should be avoided to back up into the upper abdomen during trendelenburg position.
17) Use of a vaginal apex closure device can be chosen by each surgeon.
18) The laparoscopic operation can also be performed through vagina.

3.4 Intraoperative sentinel lymph node localization biopsy:

Sentinel lymph node localization biopsy will not be used in this project.

3.5 Pelvic/Para-aortic lymphadenectomy:

The surgeon is required to perform pelvic lymphadenectomy± para-aortic lymphadenectomy in both groups of patients; however, the surgeon may also choose not to perform lymphadenectomy if the patient meets the following criteria:

1) Morbid obesity: (European and American criteria: weight over 100 pounds or BMI⩾40) BMI⩾37.5 for the Asian population; or BMI ⩾ 32.5 with two or more metabolic complications.
2) Requires blood thinning medication.
3) The patient's physical condition is not suitable for lymphadenectomy from a medical safety perspective.

3.6 Post-operative adjuvant radiotherapy or radiochemotherapy

If the postoperative pathology report suggests the presence of risk factors associated with recurrence (high-risk factors or intermediate risk factors), postoperative adjuvant therapy is required if necessary.

(1) High-risk factors: positive parametrium, positive incisional margins, andpositive pelvic lymph nodes.

Positive parametrium:externalpelvic beam radiation therapy (EBRT) + cisplatin concurrent chemotherapy. Cisplatin (DDP) 40 mg/m^2/w (week, w), or carboplatin if cisplatin is not tolerated (AUC=2). (If positive for parametrial tissue, local push of 5-10 Gy may be appropriate).

Positive vaginal incisional margins: external pelvic radiation therapy (EBRT) + cisplatin concurrent chemotherapy + vaginal backloading radiotherapy, with cisplatin (DDP) 40 mg/m^2/w, or carboplatin (AUC=2) if cisplatin is intolerant.

Positive pelvic lymph nodes: one course of systemic chemotherapy + external pelvic radiation radiotherapy (EBRT) ± extended field radiotherapy to the abdominal aorta + concurrent chemotherapy with cisplatin (40 mg/m^2/w) + 3-6 courses of adjuvant systemic chemotherapy after radiotherapy (paclitaxel + cisplatin or carboplatin preferred

OB&GY of Fudan University

for large intravenous chemotherapy regimens, can use both domestic or imported). If the pathology confirms positive abdominal aorta or positive common iliac lymph nodes, irradiation of para-aortic extended field will be performed; when the pelvic lymph nodes are positive and the pathology result of abdominal aortalymph nodes clearance is negative (if only abdominal main biopsy without clearance, positive irradiation will be performed, negative is equivalent to no clearance), no extended field irradiation will be performed; if the lymph nodes in the pelvic lymph nodes are positive and the lymph nodes in the para-aortic lymph nodes are not cleared, but the image is suscepted of metastasis in the para-aortic lymph nodes (full lymph nodes, length to diameter ratio close to 1), it is recommended to extend field irradiation.

(2) Intermediate risk factors (tumor size, interstitial infiltration, positive lymphovascular space), squamous carcinoma according to "Sedlis criteria": external pelvic radiation therapy (EBRT) + cisplatin concurrent chemotherapy, cisplatin (DDP) 40mg/m^2/w.

(3) Adenocarcinoma and adenosquamous carcinoma are treated according to intermediate risk factors, following the four-factor approach (i.e. adenocarcinoma or adenosquamous carcinoma with positive lymphovascular space, tumor ≥3 cm, and infiltration of the outer 1/3 of the cervical canal should be treated with concurrent radiotherapy). External pelvic radiation therapy (EBRT) + cisplatin concurrent chemotherapy with cisplatin (DDP) 40 mg/m^2/w, or carboplatin (AUC=2) if cisplatin is not tolerated.

(4) Positive proximal margin (negative margin ≤0.5cm), treatment as positive vaginal margin, external pelvic irradiation therapy + cisplatin concurrent chemotherapy + brachytherapy.

The EBRT implementation:

(1) Recommend to start at 4 weeks postoperatively and no later than 8 weeks. Prescribed dosage: squamous carcinoma, 45 Gy; adenocarcinoma, 50.4 Gy; conventional segmentation 1.8 Gy per session, once daily, Monday through Friday each week. Large unresectable metastatic lymph nodes are given a simultaneous additional push (SIB 2.1-2.2Gy) or sequential additional dose of 10-15Gy.

(2) Scope of irradiation: pelvic radiation field ± extended abdominal aortic field ± brachytherapy.

(3) Pelvic radiation field: at least 3-4 centimeters below the vaginal dissection, parametrial tissue and adjacent lymph node drainage areas (common iliac, internal and external iliac lymph nodes, closed foramen, presacral) must be included.

(4) Para-aortic extension field: the upper border reaches the level of the left renal vein, the left side reaches the inner edge of the psoas major muscle and the right side 3-5 millimeters around the inferior vena cava.

(5) Intensity-modulated radiation therapy (IMRT) or volumetric-modulated

0B8GY of Fudan University

arcadiotherapy (VAMT) is recommended for EBRT with weekly cone beam CT (CBCT) once a week.

The Implementation of retrovaginalradiotherapy

(1) Retrovaginal radiotherapy is started after the completion of external pelvic irradiation, twice per week. (It is recommended that the total duration of external irradiation radiotherapy + retrovaginal radiotherapy treatment does not exceed 56 days).

(2) Recommend 3-D rear-loading technique: CT or MRI localization, using columnar and ovoid applicators with ^{192}Ir-source or ^{60}Co, for high-dose-rate rear-loading radiotherapy.

(3) High-risk clinical target volume (HR-CTV): including the area 5 millimeters outside the vaginal mucosa and 1 centimeter above the vaginal stump.

(4) More than 90% of the volume of HR-CTV per session is included in the prescribed dose range (6 Gy × 3 sessions); for two-dimensional rear-loading radiotherapy plans, the prescribed dose point is 5 millimeters below the vaginal mucosa.

(5) The sum of external rectal irradiation and 2 Gy equivalent total dose (EQD2) of postoperative radiotherapy (2cc) is less than 65~75 Gy; sigmoid colon is less than 70-75 Gy, and bladder 2cc is less than 80~90 Gy.

The implementation of CCRT.

CCRT is given weekly cisplatin (DDP) intravenous chemotherapyduring EBRT. Cisplatin (DDP) 40 mg/m^2/w is recommended for the first 3 days of the first week of radiotherapy initiation, with an expected 5 cycles.

Note: SEDLIS criteria

LVSI	Depth of interstitial invasion	Tumor size(cm) (determined by clinical gynecological examination)
+	Deep 1/3	Arbitrary
+	Medium 1/3	≥2cm
+	Shallow 1/3	≥5cm
-	Medium or deep 1/3	≥4cm

LVSI: Lymphovascular space invasion

3.7 Recording of follow-up data:

3.7.1 Preoperative assessment:

a) Standard preoperative laboratory tests determined by each center: (e.g., routine blood and urine test, coagulation function, blood liver, kidney function, electrolytes, HIV, RPR,

OB&GY of Fudan University

hepatitis markers)

b) 12-lead ECG

c) Tumor indicators: SCC antigen test (SCCA) and CA-125

d) HPV typing test and TCT test in the last 3 months (only one preoperative test is required)

e) Pathology or pathology consultation at each center (LEEP or conization is required for IA1 with LVSI and IA2).

f) Imaging (CT scan of the chest, enhanced CT of the upper abdomen and pelvis, enhanced MRI to assess lesion size or PET-CT to assess retroperitoneal lymph node metastases)

g) Collection of relevant medical history, height and weight, demographic data, and health insurance-related information of the patients

h) Record all medications (including prescription drugs, over-the-counter drugs, vaccination, antibiotics, herbal medicine) used by the patient in the past 3 months

i) Sign the informed consent form

j) Record an inclusion criteria screening form

k) Complete the randomization procedure, entering randomization codes for patients and recording the results

l) Sign the informed consent form for the relevant surgery

m) Patients may scan the QR code to register for the web-based follow-up platform

[Note]:

- **Pathologic evaluation:** Each patient must have a clear histologic diagnosis of cervical squamous cell carcinoma, cervical adenocarcinoma, or cervical adenosquamous carcinoma prior to radical cervical cancer surgery. If the cervical lesion is relatively evident, although the biopsy pathology shows patches of squamous cells without clear interstitial infiltration, the clinical presentation of the lesion is consistent with invasive cervical cancer, and then the patient may still be included in the study.
- **Clinical assessment:** cervical lesion size was measured by pelvic-enhanced MRI; the presence of metastases in the parametrium was determined by physical examination of the operator; and lymph node status was assessed by pelvic-enhanced MRI in combination with other imaging such as PET-CT.

3.7.2 Documentation of the surgical procedure

a) Surgery based on randomized outcome: open or laparoscopic operation.

b) Record intraoperative and postoperative information (including details of the procedure, duration of surgery, duration of anesthesia, intraoperative bleeding, intraoperative complications, details of blood transfusion and any other special circumstances surrounding the procedure).

OB&GY of Fudan University

3.7.3 Post-treatment follow-up records

One week after the end of treatment.

a) Record postoperative pathology results and corresponding follow-up treatment plan

b) Review laboratory tests (e.g. blood and urine routine test, coagulation function, blood liver, kidney function, electrolytes, drainage fluid creatinine)

c) Postoperative indwelling catheter for 3-6 weeks and review of residual urine when the catheter is removed

d) Postoperative complications (lymphatic cysts, dysuria, vaginal bleeding)

Review every 3 months for 2 years after completion of treatment with:

a) Imaging examinations (chest CT + pelvic enhancement MRI + upper abdomen enhancement MRI or PET-CT)

b) Tumor indicators: SCC antigen test (SCCA) and CA-125

c) HPV and TCT test (once a year after completion of treatment)

d) Adverse events recording

e) Assessment of quality of survival and quality of sexual life using the Quality of Survival Scale for Cancer Patients (EORTC QLQ-C30 v3.0), the Cervical Cancer Sexual Life Scale (EORTC QLQ-CX24), and the Patient Reported Clinical Outcome Assessment Scale for Cervical Cancer PRO147 (V1.0).

After 2 years, the study will be reviewed every 6 months until the end of the trial. The review will includ gynecological examination, tumor indicators (SCC antigen detection (SCCA) and CA-125), TCT examination and HPV examination, imaging (chest CT + pelvic enhancement MRI + upper abdomen enhancement MRI or PET-CT), and adverse event records.

3.8 Criteria for Patient Withdrawal

Patients have the right to withdraw from the trial treatment or study at any time without any reason. The investigator also has the right to withdraw a patient from the trial treatment or study in the event of secondary disease, adverse events, protocol violations, administrative reasons, or other reasons. Participation in the study, whether or not the affected patient participates, will not affect the relationship between the patient and the physician and will not result in a loss of medical or other benefits to the patient. However, in such cases, the investigator will make appropriate efforts to determine and document the reasons for the patient's voluntary withdrawal from the study. If a patient withdraws from the study, a complete final evaluation should be conducted at the time of patient withdrawal. This will be reported along with the study results and all attempts to locate patients lost to follow-up will also be documented.

Patients will be seen by their physician if they withdraw from the study. According to routine gynecologic oncology protocols, they should continue to receive care and treatment from an experienced physician until further follow-up is deemed unnecessary

OB&GY of Fudan University

by the treating physician.

Patients will be withdrawn from the study under the following circumstances:

1) Study completion/termination, or death
2) Patient demands withdrawal
3) Patient is unable or unwilling to cooperate with follow-up
4) Patient has other serious, non-surgical complications
5) Patient's follow-up information cannot be obtained
6) The study is terminated or cancelled at the request of the sponsor
7) Other relevant reasons

If a patient moves to another treatment center and requires changing of physician, the investigator will make a reasonable attempt to locate that center's physician and request cooperation in order to complete the follow-up.

In many cases, patient withdrawal from the study constitutes discontinuation of treatment and/or discontinuation of completion of related forms, such as quality of life. In these cases, the investigator should obtain the patient's permission to continue monitoring their disease status (relapse, survival, toxicity, etc.) through the patient's record.

Excessive exit rates can make a study uninterpretable, and therefore, unnecessary subject dropouts should be avoided.

3.9 Combination and simultaneous treatment:

Supplementary therapy will be administered according to the postoperative pathological findings of the patients. Those with high-risk factors who have positive parametrium will be supplemented with external pelvic irradiation therapy + cisplatin/carboplatin concurrent chemotherapy, those with positive vaginal margins will be selected for external pelvic irradiation therapy + retrovaginal radiotherapy + cisplatin/carboplatin concurrent chemotherapy, and those with positive pelvic lymph nodes will be treated with 1 course of systemic chemotherapy + external pelvic irradiation radiotherapy ± extended field radiotherapy adjacent to the abdominal aorta + cisplatin concurrent chemotherapy + 3-6 courses of adjuvant systemic chemotherapy after radiotherapy. Those with intermediate risk factors will be treated with external pelvic irradiation radiotherapy + cisplatin concurrent chemotherapy, and those with positive proximal margins will be treated with external pelvic irradiation radiotherapy EBRT + cisplatin concurrent chemotherapy + retrovaginal radiotherapy. Targeted immunotherapeutic agents such as bevacizumab and PD-1, which have just obtained some results in clinical studies, are not considered in this trial for the time being. It could be used according doctor’s orders.

3.10 Outcome measurements:

OB&GY of Fudan University

1) Main outcome indicators: 5-year OS.

2) Secondary outcome measurements: 5-year PFS, refer to RECIST criteria.

3) Other outcome measurements: operation time, anesthesia time, intraoperative bleeding volume, intraoperative complications, postoperative complications, postoperative pain score, postoperative hospital stay, one-month postoperative survival quality, one-year postoperative survival quality, sexual quality of life.

4) Other evaluation parameters: patient's demographic characteristics, signs and symptoms, stage, pathological type, tumor site, comorbidities, medical history, length of hospital stay and cost.

5) This study intends to sample and collect intraoperative tumor tissues from tumor patients and evaluate specific tumor stages with genetic testing to guide later treatments such as targeted or immune therapies. Histological studies and molecular studies will also be conducted.

	Pre-phase	0-12 months	12-24 months	24-36 months	36-60 months
Meet the researchers	X				
Obtain IRB approval	X				
Funding application	X				
Enrollment of study patients		X	X	X	
Data monitoring		X	X	X	X
Data collection		X	X	X	X
Data analysis					X
Summary and discussion					X

3.11 Subjective schedule

OB&GY of Fudan University

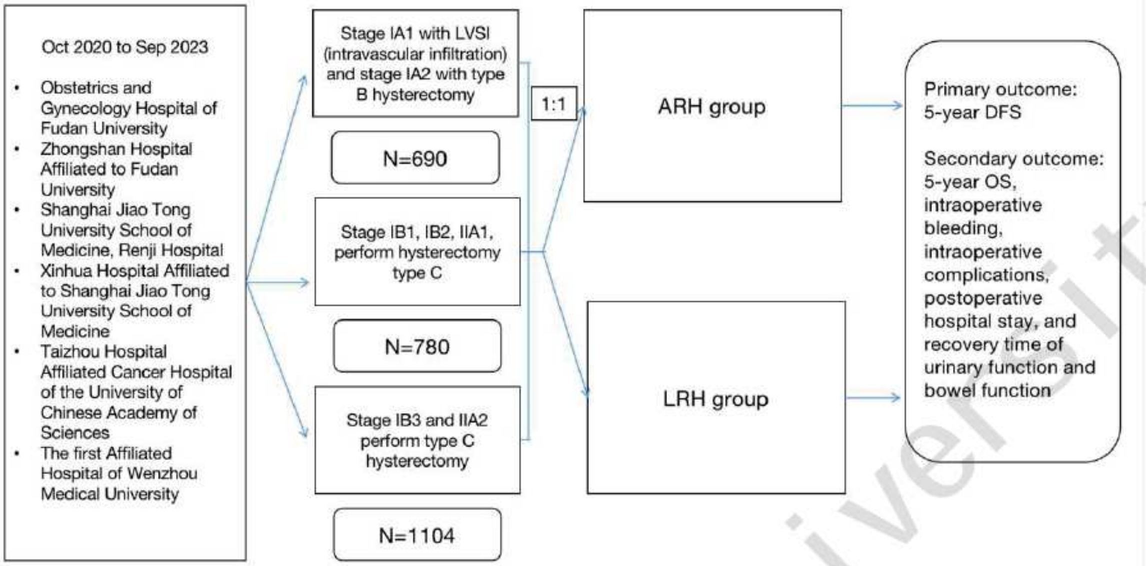

Note: For patients with stage IB1, IB2, IIA1, IB3, and IIA2, the surgical method will be selected according to the maximum diameter of the mass. C1 type hysterectomy will be used for diameters <2 cm, C2 type hysterectomy will be used for diameters >4 cm, and C1 type or C2 type hysterectomy will be selected by the surgical team members according to the actual intraoperative condition for diameters between 2 cm and 4 cm.

3.12 Sample size

This study uses a non-inferiority trial design, and the primary outcome is the rate of OS at 5 years. The sample size is calculated based on the difference between the two groups of 5-year OS rate. The 5-year OS rate of the patients in the open surgery group is estimated to be 72% based on the previous clinical data from our hospital.

Based on the assumptions of (i) the 5-year OS rate of 72% in the open surgery group, (ii) a non-inferiority margin of 8%, (iii) 80% power, and (iv) a one-sided alpha of 0.025, the sample size estimation resulted in 495 subjects per group (calculated with SAS software, version 9.4). Considering a 10% drop-out rate and the randomization scheme with block size of 6, a total of 1104 subjects should be randomized (552 per group).

3.13 Recruitment methods:

The study team centers will advertise this study at their cervical cancer clinic. If the patients are interested, they will be further introduced in detail about the study background, randomization and surgical procedures, and the patients can voluntarily choose whether to participate in the trial. After patients sign the informed consent forms, members of the study team will review their eligibility according to the inclusion and exclusion criteria. Patients will then be randomized to receive either of the surgical

OB&GY of Fudan University

procedures. Finally, the patients will be followed up according to the follow-up schedule.

IV. Allocation, Blinding and Unblinding

4.1 Allocation of the interventions

4.1.1 Allocation sequence generation: In this study, a centralized block randomization with a block size of 6 will be used to randomly assign patients to the laparoscopic surgery group or the open surgery group at a 1:1 ratio. The randomization scheme will be completed by an independent statistical team at the Clinical Trial Center of the Children's Hospital of Fudan University by creating and sequencing random seed numbers (SAS 9.4 software). The intervention protocols determined by the random assignment sequence will be in sequentially numbered, non-transparent, sealed envelopes by zone: each of the 6 subgroups for each zone will be placed sequentially in six small, non-transparent, sealed envelopes numbered from 1 to 6, and then uniformly placed in the same large envelope marked with the corresponding zone number. The randomization assignment will be administered by the central coordinator at the Obstetrics and Gynecology Hospital of Fudan University who is not involved in the implementation of the intervention or the outcome observation. The enrollment is competitive, and the sub-center coordinator will contact the central coordinator before the inclusion of the first patient in each group. The central coordinator will follow the randomization scheme and assign the large envelopes with the group numbers to each center following the order of contact. Each sub-center will open the envelopes according to the order of signing the informed consent form, and strictly follow the numbered order of the envelopes, and then inform the participating physicians of the assignment scheme and complete written registration form.

4.1.2 Allocation concealment mechanism

1) In this study, the surgical procedures will be selected in a randomized manner, and the CTU members will place the intervention modality in an envelope and give it to each center's coordinator who is not involved in the implementation of the intervention or the observation of the outcome to manage the allocation. The envelope will be opened by the investigators at each center following the enrollment order, to realize blinded preoperative randomization.

2) Data will be entered by dedicated study staff, who will be blinded to the randomization allocation and surgical procedure.

4.1.3 Assignment implementation (who generates the serial numbers, who recruits the subjects, and who assigns them)

1) The surgeons will perform the recruitment and obtain informed consent, and perform the surgical procedure.

2) The CTU team designs the randomization method and generates the corresponding

OB&GY of Fudan University

envelopes.

3) The allocation of surgeries according to the order of informed consent signed by the study participants in each center according to the randomization method to achieve an equal distribution of reporters in the open and lumpectomy groups.

4) The randomization staff will record the randomization numbers and procedure.

5) Follow-up of each patient and recording of relevant data and adverse events will be performed by dedicated follow-up personnel.

6) Data entry into the RedCap database will be performed by dedicated data entry personnel.

7) The CTU and the principal investigator will perform statistical analyses.

4.2 Blinding

4.2.1 Who to blind and how to implement it

1) The specific procedure will not be known to the surgical team of each center until after the patients have been randomized during the administration phase of the intervention.

2) The randomization process will not be known to data entry personnel after the operation.

3) Statisticians will also be unaware of the randomization process.

4) The randomization process is also unknown to the personnel responsible for endpoint evaluation.

4.2.2 Unblinding

1) The total randomization form will be kept exclusively by the dedicated staff at each center, and a letter which contains information of the group assignment and the surgical method will be set up for each group with the number.

2) The unblinding will be conducted to reveal the information of each patient with corresponding surgical procedure, only after final data analyses.

3) Since this study does not involve drug use, there is no emergency unblinding process in place.

V. Methods of data collection, management and analysis

5.1 Data collection and patient retention

5.1.1 Protocols for estimating and collecting outcome indicators, baseline and other trial data

Based on the study protocol and data analysis plan, a case report form (CRF) has been developed and validated. Required fields are set to ensure the completeness of key data. The CRF will be completed by trained investigator at each site, and each enrolled case has to complete the CRF, which will be reviewed by the investigator and handed over to data manager for data entry and management at each site.

OB&GY of Fudan University

5.1.2 Improve patient engagement and completion of follow-up, including outcome data to be collected for patients who withdraw or change treatment methods
1) Detailed patient orientation by study team members at each center during the recruitment phase to achieve fully informed patients.
2) The study team members will provide long-term remote condition assessment services and related information consultation services for all study patients.
3) Patients will be followed up regularly by dedicated staff to reduce the rate of loss to follow-up.
4) The follow-up system will regularly remind the subjects of the follow-up time and the follow-up items according to the follow-up nodes.

5.2Data management (entry, coding, encryption and storage)

5.2.1 Entry and coding protocols:
A database for data management and entry is designed based on the finalized CRF form. This study uses RedCap, which was developed by a professional CRO data management team, and the project data entry and management will be handled by dedicated personnel. To ensure the accuracy of the data, double entry should be performed by two research members independently, and blind verification and statistical analysis should be performed after software verification and manual error correction. The database will be locked after accuracy confirmation. The locked data files then will be submitted to an independent third-party statistical team for analysis. The statistical analysis plan (SAP) is developed by the statistical team during the study subject inclusion process, and finalized after repeated discussions with the investigator team, and uploaded to the clinical trial registry website before the last patient data collection is completed at the study registry website, or submitted for publication with the protocol.

5.2.2 Data storage and access
All information on research subjects is collected and stored in locked cabinets by dedicated personnel, and access to it is only possible with the relevant viewing privileges.

5.3 Statistical methods

5.3.1 Outcome statistical methods

Statistical analysis of the study will be undertaken by the CTU of the Children's Hospital of Fudan University. Interventions began at the study design stage, based on key scientific questions, clear design protocols, and statistical analysis methods based on the properties of the primary outcome indicators, whereby sample sizes were calculated based on expected effects. After study inclusion begins until the last patient data collection is completed, a detailed SAP is written and uploaded to the Clinical Trials Registry website. The following is an overview of the statistical analysis strategy and methods.
All analyses will be performed on an intention-to-treat (ITT) basis, except for sensitivity

OB&GY of Fudan University

analysis that will be performed according to per-protocol (PP) treatment.

5.3.2Analysis of primary outcome data

a) This study is non-inferiority trial design, and the primary outcome is the rate of OS at 5 years. The hypothesis is as follows:

Hypothesis: Let $\pi 1$ denote the rate of OS at 5 years in the open surgery group, and let $\pi 2$ denote the rate of OS at 5 years in the minimally invasive surgery group, the non-inferiority margin =8%. The null hypothesis is H0: $\pi 1 - \pi 2 \geq$ versus the alternative hypothesis is H1: $\pi 1 - \pi 2 <$.

b) The curves of OS at 5 years will be estimated using the Kaplan-Meier method. The logrank test will be used to test the above hypothesis, the 5-year OS rate difference and its 95% confidence interval (CI) for the comparison between the two groups will be estimated. The minimally invasive surgery will be considered non-inferior to the open surgery if the one-sided 95% upper limit is less than , where = 8%, the predetermined non-inferiority margin.

c) Covariate adjusted analysis:

An analysis of the primary outcome adjusting for the blood loss during operation, operative duration, and postoperative pain score will be performed using Cox proportional hazards regression model. The hazard ratio of 5-year OS and 95% CI will be estimated.

d) Subgroup analysis:

We will stratify by tumour staging, and perform Cox proportional hazards regression model to estimate the hazard ratio and 95% CI of the 5-year OS.

e) Sensitivity analysis:

A sensitivity analysis of the primary outcome will be performed using the PP strategy.

5.3.3 Secondary outcome analysis

a) The rate of PFS at 5 years:

Cox proportional hazards model will be used to estimate the hazard ratio and 95% CI for the effect of treatment on the 5-year PFS rate.

b) Analysis of continuous outcomes:

The continuous outcomes include operative duration, anesthesia time, blood loss during operation, postoperative pain score and postoperative hospital stay. The outcomes with normal distribution will be summarised using mean and standard deviation (SD), while the outcomes with non-normal distribution will be summarised using median and interquartile. The differences in the outcomes and 95% CIs will be analysed by generalised linear model (GLM) with treatment as fixed effect and with normal distribution and identity link function.

c) Analysis of binary outcomes:

The intraoperative complications, postoperative complications, one-month and one-year postoperative quality of life and sexual life will be treated as binary outcomes, and will

OB&GY of Fudan University

be summarised by number (%) of participants with the event. The differences in the outcomes and 95% CIs will be analysed by GLM with treatment as fixed effect and with binomial distribution and identity link function.

d) Interim analysis

The O'Brien Fleming method will be used to conduct an interim analysis three years after the study. If the study has conducted only once interim analysis, the α level will be set as 0.048 in the final analysis.

4.Safety analysis

Adverse events (AEs) will be summarised using the number of AEs, the number (%) of participants with AEs by groups.

5.All analyses will be performed with a two-sided significance level of 0.05 and conducted using SAS software, version 9.4 (SAS Institute).

5.3.4 Definition of non-compliant population, and analysis and management of missing data

All data will be monitored by an independent data monitoring committee consisting of surgical experts, epidemiologists, RCT experts, statisticians and surgeons.

5.3.5 Statistical analyses

All statistical analyses will be performed with a two-sided significance level of 0.05 and conducted using SAS software, version 9.4 (SAS Institute).

VI. Quality of life questionnaire

6.1 Patient-reported clinical outcome assessment scale PRO 147 (V1.0) for Cervical Cancer

The research team developed its own scale assessment related to patient reported outcomes in cervical cancer, which is based on the patient's perspective to assess the outcome after surgery. Cervical cancer is the fourth most common cancer in women, and its incidence has been on the rise in recent years, especially in younger patients. Since the uterus is located in the central pelvis, damage to pelvic floor tissues by surgery may cause a variety of complications such as urinary, intestinal, vaginal and lower limb dysfunction, which cannot be accurately assessed by conventional scales. Considering the high survival rate of patients with early-stage cervical cancer, it is especially important to study the short-term and long-term treatment effects for patients.

According to the U.S. Food and Drug Administration (FDA), patient reported outcome (PRO), a measure of any aspect of a patient's health status that comes directly from the patient, is a valid patient-centered, multifaceted means of evaluating treatment outcomes (such as symptom improvement or related function) that is widely used in clinical practice and trials.

OB&GY of Fudan University

The research team has developed the Patient-reported Clinical Outcome Assessment Scale PRO 147 (V 1.0) for cervical cancer based on this concept. This scale is based on three domains including daily life, psychological and social life, with 147 questions covering 13 latitudes: urinary dysfunction (URI), bowel dysfunction (BOW), pelvic organ prolapse (POP), loss of ovarian function (OVA), lymphatic dysfunction (LYM), neurological dysfunction (NEU), sexual dysfunction (SEX), anxiety (ANX) depression (DEP), stigma (SHA), life beliefs (BEL), social support (SUP), and daily life (LIF) to assess patient prognosis. At present, our scale has been screened, and completed the expert review, and now is in the pilot study phase. So far, there are about 100 patients have completed the survey, and the information is very useful for future complication management and surgical efficacy assessment.

6.2 Generic quality of life questionnaire

Other selected quality of life questionnaires relate to postoperative symptoms (e.g., pain) as well as disease-specific and general health-related quality of life. These surveys are self-administrated and provide an understanding of the patient's quality of life, and will take less than 30 minutes to complete.

The current scale used for study follow-up is mainly the EORTC Quality of Life Measurement Scale QLQC30 (V3.0) scale, which is intended for most oncology patients and is a universal scale. From a systematic review including 156 studies, Casper Tax et al. found that the European Organization for Research and Treatment of Cancer, Quality of Life Questionnaire, Cervical Cancer Module (EORTC QLQ-CX24) and the Functional Assessment of Cancer Therapy for Cervical Cancer (FACT-Cx) are the only cervical cancer-specific instruments to date. EORTC-QLQ-CX24 appears to be the most commonly used instrument to assess Health Related Quality of Life (HRQoL) in patients with cervical cancer, and the developers of the EORTC QLQ-CX24 proposed in their study that no test-retest analysis was performed and response over time. Both scales are simple and universal, but can only distinguish between patients with early versus advanced disease and those who are receiving versus not receiving treatment. However, whether the efficacy of different treatments (e.g., surgery versus chemotherapy) or different surgical approaches (e.g., laparoscopic radical hysterectomy versus abdominal radical hysterectomy) can be distinguished has not been reported. It indicates that EORTC-QLQ-CX24 has some limitations in the evaluation of efficacy.

VII. Methods of Data Monitoring

7.1 Study monitoring

7.1.1 Trial Safety Committee

An independent Trial Safety Committee (TSC) will be established to review the safety and efficacy data collected during the study. The TSC will be composed of individuals who are independent of the study and not directly or indirectly involved in the

OB&GY of Fudan University

administration of the study. Members will include the following individuals.

Two gynecologic oncologists not involved the trial.

Two statisticians not involved in the trial.

Members of the Gynecologic Oncology Committee (Shanghai Medical Association Obstetrics and Gynecology Section, Shanghai Physicians Association Obstetrics and Gynecology Section)

7.1.2 Trial Management Committee

A Trial Management Committee (TMC) will be established for this study to review and oversee data and analysis. The TMC will be composed of the main center study leader, the sub-center leaders, and the study statistician, and will not have a conflict of interest with the funding provider. The management committee will meet every four months, and after each meeting, the committee will recommend that the study continue according to the protocol or recommend modifications to the protocol based on the results of the data review.

7.1.3 Data Monitoring Committee

A Data Monitoring Committee (DMC) will be established to independently review and oversee data and analysis. The DMC does not have a conflict of interest with the funding agency.

7.2 Interim Analysis

The ethics committee has the right to terminate the study when the number of deaths reaches a critical level or when the difference in progression-free survival or overall survival between the two groups is significant; the ethics committee has the right to terminate the study when there is a violation of ethics during the progress. To ensure the compliance of the patients, participants in this study will be given priority for treatment at our hospital, and there will be dedicated staff for post follow-up and data collection.

7.3 Quality Control

The study process will be closely followed by the project sponsor, the sub PIs of each study center, the CRO manager, and the surgical team. Data quality is monitored by the independent DMC, CRO project manager, and the surgical team.

Clinical observation and follow-up: surgical treatment plan, surgery-related indicators, intraoperative and postoperative complications, postoperative pathology records, postoperative adjuvant treatment including radiotherapy and chemotherapy dose and time, number of courses and adverse reactions; follow-up will be conducted every three months for 2 years after surgery; then every 6 months from year 3 to year 5 after surgery.

Measures to ensure patients' compliance: patients participating in this study will be given priority in our hospital for treatment, and post-operative follow-up will also be

OBGY of Fudan University

assigned.

VIII. Definition and treatment of adverse events

8.1 Definition of adverse events

The Adverse Event (AE) is an adverse medical event that occurs after a patient or a subject in a clinical trial receives a drug, but is not necessarily causally related to the treatment.

Any adverse medical event that occurs in this trial from the time the patient signs the informed consent and receives the treatment until 1 month after the end of the treatment, regardless of whether it is causally related to the surgery performed, is judged as an adverse event, including symptoms, signs, diagnosis, and abnormal laboratory tests.

8.2 Observation, recording and treatment of adverse events

Observation and recording: the investigator should carefully observe any adverse events occurring in the patient during the study, and require the patient to truthfully reflect the changes in condition after treatment and avoid eliciting questions. Pay attention to adverse reactions or unanticipated toxicities (including symptoms, signs and laboratory tests) while observing side effects. Regardless of whether the adverse event is related to the surgery performed, it should be recorded in detail in the CRF, including the time of occurrence of the adverse reaction, symptoms, signs, extent, duration, laboratory test indicators, treatment methods, process, results, follow-up time, etc. The combined use of drugs should be recorded in detail for analysis of adverse event and test drug correlation, and the record should be signed and dated.

Medical treatment of patients: When adverse reactions are reported, the investigator should take necessary treatment measures according to the condition, such as dose adjustment, temporary interruption of medication, etc., and decide whether to terminate the trial. The occurrence of serious adverse events, the unit undertaking the pilot study must immediately take the necessary measures to ensure the protection of the safety of the subject.

The first safety analysis will be conducted after 20 patients have completed treatment. TSC will review all safety data collected during the study twice a year.

IX. Cost-benefit Analysis

We will also assess the subjects' household income and sources of surgical treatment costs, record their health insurance reimbursement rates, and evaluate the impact of different health insurance rates on patients' household burden.

X. Review

The main center at the Obstetrics and Gynecology Hospital of Fudan University will closely monitor the study. The monitor will maintain an effective understanding of the

OB&GY of Fudan University

study through observation, review of study records and source documents, and discussions with investigators and staffs about the conduct of the study. The main center or its designee will carefully monitor all aspects of the study to ensure that the study complies with applicable government regulations regarding current good clinical practice and current standard operating procedures.

Investigators and institutions involved in the study will allow trial-related monitoring, review, Institutional Review Board (IRB) or Human Research Ethics Committee (HREC) reviews and regulatory inspections through direct access to raw data/documents.

XI. Ethics and Dissemination

11.1Ethical approval

A total of 1104 patients will be recruited in six centers: Obstetrics and Gynecology Hospital of Fudan University, Zhongshan Hospital affiliated to Fudan University, Xinhua Hospital affiliated to Shanghai Jiao Tong University School of Medicine, Renji Hospital affiliated to Shanghai Jiao Tong University School of Medicine, Taizhou Hospital Affiliated Cancer Hospital of the University of Chinese Academy of Sciences, and the First Affiliated Hospital of Wenzhou Medical University. All patients will be fully informed of the purpose, methods, benefits and potential risks of this clinical trial and sign the informed consent form.

The study protocol has been sent to each sub-center and has been reviewed and approved by the ethics committees of respective hospitals.

11.2 Study protocol modification plan

The main objective of this study is to observe PFS and OS after cervical cancer surgery. The patient sample size is large and the enrollment time is long, so it needs to take more than 3 years to complete the enrollment. Major surgery, postoperative treatment and initial follow-up could be completed during this period. It is expected that initial outcomes can begin to be produced after 3 years. However, tumor follow-up will require at least 5 years to obtain complete and comprehensive data, especially for PFS and OS. Previous similar studies have taken about 10 years to reach a final and convincing conclusion. After the initial 3 years of funding for this study, there is still numerous and long-term workloads that need to be completed. As a result, it is expected that regular follow-up and analysis will be needed for several years after the current grand ends. Therefore, we hope that the time frame for the use of funds and the time frame for the publication of results can be extended to facilitate the long-term implementation of the project and the final interpretation of the research results. If the project does not complete the study objectives or the task acceptance conclusion is false, the project lead organizations should return the outstanding funds.

OB&GY of Fudan University

11.3 Informed consent

All patients have been fully informed of the purpose, methods, benefits and potential risks of this clinical trial and should sign the informed consent form (see Appendices for details).

Written informed consent should be obtained from each patient before enrollment. A template of informed consent will be provided by the main center. Prior to recruitment and enrollment, each prospective patient will be given details of the study and enough time to understand the approved informed consent form. The investigator will inform the patient of the study purpose, the randomization procedure and the follow-up schedule. The investigator will discuss the potential benefits and risks. The investigator will inform the patient that her medical records will be reviewed by the Shenkang Hospital Administration Center and Hospital Department.

Patients will be informed by the investigator that they are free to decline participation, and if they choose to participate, they may withdraw at any time without affecting further medical care.

This study has complied with the Regulations of the People's Republic of China on Human Genetic Resources Management in the collection, storage and utilization of biological specimens and genetic resources (see Appendices for details).

XII. Confidentiality

All medical record, relevant laboratory data, completed CRF forms, and protocol-related documents will be kept in a special locked cabinet for each study participant, and access to them will be granted only after providing relevant permissions.

XIII. Declaration of Competing Interests

The protocol designer and all study participants have no relevant ties or interests with the manufacturers of the corresponding drugs, devices, etc. and fund providers.

XIV. Data Collection

Relevant experimental data and basic information about the study subjects will be collected and filled out by the surgical team, and follow-up information will be collected by the follow-up staff and enter into the RedCap system by designated research staff, which were accessible to the project team members but did not know the corresponding information.

Each investigator and organizations in this study need to sign the "Project Data Submission Undertaking" (see Appendices for details), and commit to the standardized operation of data collection, submission and disclosure.

OB&GY of Fudan University

XV. Affiliation and Post-test Care

The surgical procedures performed in this protocol are those that have been in use for more than 10 years and meet international NCCN guideline standards, with no operational abnormalities and no corresponding terms attached.

XVI. Dissemination Policy

The intellectual property rights of this study, include patents, copyrights, trademarks, copyrights of computer software, technical secrets, and trade secrets that abiding by the law. New intellectual property rights generated in the course of this study belong to the main center, which is responsible for declaring and enjoying priority, and the sub-centers are ranked according to their contribution. Intellectual property rights resulting from the independent completion of the research work may be owned by each center independently. No party shall disclose to other parties without the consent of the collaborating party.

The main center has the right to use all the information and data, and the sub-centers have the right to use the information and data of their center. The main center is responsible for statistical processing and analysis, writing summary reports and papers. The authorship will be determined by the main center, based on the contribution (number of valid cases enrolled), and the results will be shared. For collective data and collective results, the main center will be the first author and corresponding author, and sub-centers will be assigned co-first author or co-corresponding author or co-author based on their contributions. The publication of each center's respective data will be signed by each center. Results published separately by sub-centers are required to include the main center as a co-corresponding author. The sub-center cannot publish the results related to this study in any form until the main center has published the main results of this study. All personnel of each center cannot duplicate or cross-report this project. The "Three-Year Action Plan Major Clinical Research Project" and the project approval number are indicated.

XVII. Appendices

1. Informed Consent Form
2. Commitment to the Regulations of the People's Republic of China on Human Genetic Resources Management
3. Commitment to Submit Project Data
4. Statement of not involving the use of highly pathogenic microorganisms

OB&GY of Fudan University
